# Protein production dynamics and physiological adaptation of recombinant *Komagataella phaffii* at near-zero growth rates

**DOI:** 10.1186/s12934-024-02314-3

**Published:** 2024-02-08

**Authors:** Corinna Rebnegger, Benjamin L. Coltman, Viktoria Kowarz, David A. Peña, Axel Mentler, Christina Troyer, Stephan Hann, Harald Schöny, Gunda Koellensperger, Diethard Mattanovich, Brigitte Gasser

**Affiliations:** 1 CD-Laboratory for Growth-Decoupled Protein Production in Yeast at Department of Biotechnology, University of Natural Resources and Life Sciences (BOKU), Vienna, Austria; 2 Department of Biotechnology, Institute of Microbiology and Microbial Biotechnology (IMMB), University of Natural Resources and Life Sciences, Vienna, Muthgasse 18, 1190 Vienna, Austria; 3 Austrian Centre of Industrial Biotechnology (ACIB GmbH), Muthgasse 11, 1190 Vienna, Austria; 4 Department of Forest- and Soil Sciences, Institute of Soil Research, University of Natural Resources and Life Sciences, Vienna, Peter-Jordan-Straße 82, 1190 Vienna, Austria; 5 Department of Chemistry, Institute of Analytical Chemistry, University of Natural Resources and Life Sciences, Vienna, Muthgasse 18, 1190 Vienna, Austria; 6 Department of Analytical Chemistry, Faculty of Chemistry, University of Vienna, Waehringer Straße 38, 1090 Vienna, Austria; 7 Vienna Metabolomics Center (VIME), University of Vienna, Althanstraße 14, 1090 Vienna, Austria

## Abstract

**Background:**

Specific productivity (q_P_) in yeast correlates with growth, typically peaking at intermediate or maximum specific growth rates (μ). Understanding the factors limiting productivity at extremely low μ might reveal decoupling strategies, but knowledge of production dynamics and physiology in such conditions is scarce. Retentostats, a type of continuous cultivation, enable the well-controlled transition to near-zero µ through the combined retention of biomass and limited substrate supply. Recombinant *Komagataella phaffii* (syn *Pichia pastoris*) secreting a bivalent single domain antibody (VHH) was cultivated in aerobic, glucose-limited retentostats to investigate recombinant protein production dynamics and broaden our understanding of relevant physiological adaptations at near-zero growth conditions.

**Results:**

By the end of the retentostat cultivation, doubling times of approx. two months were reached, corresponding to µ = 0.00047 h^−1^. Despite these extremely slow growth rates, the proportion of viable cells remained high, and de novo synthesis and secretion of the VHH were observed. The average q_P_ at the end of the retentostat was estimated at 0.019 mg g^−1^ h^−1^. Transcriptomics indicated that genes involved in protein biosynthesis were only moderately downregulated towards zero growth, while secretory pathway genes were mostly regulated in a manner seemingly detrimental to protein secretion. Adaptation to near-zero growth conditions of recombinant *K. phaffii* resulted in significant changes in the total protein, RNA, DNA and lipid content, and lipidomics revealed a complex adaptation pattern regarding the lipid class composition. The higher abundance of storage lipids as well as storage carbohydrates indicates that the cells are preparing for long-term survival.

**Conclusions:**

In conclusion, retentostat cultivation proved to be a valuable tool to identify potential engineering targets to decouple growth and protein production and gain important insights into the physiological adaptation of *K. phaffii* to near-zero growth conditions.

**Supplementary Information:**

The online version contains supplementary material available at 10.1186/s12934-024-02314-3.

## Introduction

The market for biopharmaceutical proteins is valued at US $271 billion for 2021 and is expected to continue to grow in the upcoming years [1]. For the production of biopharmaceutical proteins and other recombinant proteins employed in medicine, research, and industry, budding yeasts like methylotrophic *Komagataella phaffii* (*Pichia pastoris*) have gained increasing popularity. They combine benefits of prokaryotic expression systems, including ease of genetic manipulation, short strain development times, fast growth to high cell densities, and minimal media requirements, with the ability to perform most post-translational modifications and secrete the recombinant protein into the supernatant, facilitating downstream processing [2–4].

In *K. phaffii* and other yeasts, the production of heterologous proteins is generally growth-rate-dependent, typically peaking at intermediate or near maximum specific growth rates (μ) [5, 6]. However, the respective relationship between the biomass-specific product formation rate (q_P_) and μ for a certain recombinant protein can vary greatly according to the regulatory properties of the respective promoter selected for controlling recombinant gene transcription (as well as culture conditions) and needs to be determined empirically. Multiple omics studies have demonstrated that variations in μ are readily reflected by alterations in the transcriptome, proteome, and metabolome of yeasts [7–12]. The main trend of adaptations appears to be well-conserved among different yeast species. These include the upregulation of essential genes with a role in gene transcription and protein biosynthesis at higher growth rates, while the expression of regulatory genes involved in stress response, signaling, and proteolytic degradation is elevated at lower μ. Additionally, transcriptional regulation of many processes related to the secretory pathway, such as protein folding and translocation, is positively correlated with μ [10, 13]. On a metabolic level, fast (but fully respiratory) growth in *S. cerevisiae* has been demonstrated to be accompanied by elevated amino acid pools as well as an increase in glycolytic, pentose-phosphate pathway, and TCA metabolites [11]. The increased productivity at high μ and concordantly rich nutrient conditions agrees well with the respective global rewiring of yeast cells. However, biotechnological production processes (that is, mainly fed-batch cultivation) often need to be operated at lower-than-optimal μ and therefore less-than-optimal productivity. Biomass-specific protein secretion rates of yeasts are usually substantially lower than those achieved by mammalian cell lines [14]. To compensate, yeast-based processes are typically operated at higher cell concentrations, where either mixing, aeration, or cooling capacity ultimately become the limiting aspects for process design [15, 16]. Furthermore, at higher μ, most of the substrate consumed by the cells is invested in the formation of new biomass, making biomass the most abundant (and typically undesired) by-product. Hence, partial, or ideally, full decoupling of product formation and growth would allow for more efficient production processes and therefore represents an important milestone in the quest for advancing yeast production systems.

In contrast to the vast body of research dedicated to investigating cell physiology and recombinant protein production dynamics at intermediate or higher growth rates, knowledge of the production dynamics of extremely slow or even non-growing yeast remains very scarce. Indeed, extremely slow-growing *S. cerevisiae* cultures have been explored in a non-energy-limited retentostat for the production of succinic acid [17], an important precursor for various chemicals. The non-conventional yeast *Starmerella bombicola*, a natural producer of microbial biosurfactant sophorolipids, was tested for continuous production of bolaform sophorolipid in a similar set-up [18]. However, products like proteins that require a net input of ATP for their synthesis and transport pose a greater challenge [19]. Consequently, microbial production of recombinant proteins at near-zero growth in retentostat cultivation has so far only been attempted in the filamentous fungus *Aspergillus niger* [20].

A well-controlled transition from faster growth rates to theoretically zero growth can be realized via retentostat cultivation, a form of continuous cultivation with full biomass retention. Retentostats are typically initiated from steady-state chemostat cultures. In cases where the energy (and usually also carbon) source is limiting, the respective specific nutrient uptake rate (q_S_) asymptotically approaches the specific maintenance energy requirement (m_S_), resulting in a scenario where the cells remain in a metabolically active but non-growing state [19]. Aerobic, glucose-limited retentostat cultivation as implemented for *S. cerevisiae*, *Ogataea parapolymorpha*, and *K. phaffii* relies on a gradual decrease of the initial glucose concentration in the feed to a lower setpoint using a mixing vessel upon the switch from the chemostat to the retentostat phase. This setup is specifically beneficial for retentostat cultivation of microbes that exhibit a very low m_S_, such as *O. parapolymorpha* and *K. phaffii* [21, 22], for which near-zero growth conditions (μ below 0.001 h^−1^) can otherwise only be reached after impractically long time periods (described in Vos et al. [23]). Previous retentostat cultivations of *K. phaffii* were only possible when using a *FLO8*-deficient strain, which shows decreased cell adherence to surfaces and is additionally abolished of pseudohyphal growth [21, 24]. Flo8 is a transcriptional master regulator of dimorphism and cell adherence, and most *S. cerevisiae* laboratory strains are *FLO8*-deficient. In this previous characterization of the non-producing strain at extremely slow growth, it was demonstrated that *K. phaffii* decreases its glucose expenditure on maintenance threefold from 10.0 ± 2.3 to 3.1 ± 0.1 mg g^−1^ h^−1^ with decreasing μ, unlike *S. cerevisiae*, which exhibits a growth rate-independent m_S_ [21, 23]. Interestingly, growth rate-dependency of m_S_ was recently also revealed for *O. parapolymorpha* [22].

To test the ability of *K. phaffii* for recombinant protein production and secretion at extremely slow growth, as well as to obtain insights into the physiological adaptations, we conducted aerobic retentostat experiments employing *K. phaffii* CBS2612 *Δflo8* secreting a recombinant antibody protein. Retentostat cultures were analyzed for their growth dynamics, viability, protein secretion capabilities, macromolecular biomass composition, lipidome composition, and transcriptional adaptation patterns. To obtain a more comprehensive picture of the cellular behavior at fast and very slow growth rates, the retentostat data were combined with data from separate chemostat cultivations operated at a dilution rate of 0.1 h^−1^.

## Results

### Determination of growth and production parameters of recombinant protein-secreting *K. phaffii*

Choosing a suitable promoter is essential for recombinant protein production at extremely slow growth rates [20]. The ideal candidate should allow for high levels of recombinant gene expression across a wide range of µ and maintain high transcriptional activity in progressing retentostat cultures. Based on microarray transcriptome data from the previous retentostat study using non-producing *K. phaffii* CBS7435 *Δflo8* [21] and the recently discovered beneficial effect of the *Δflo8* mutation on induction strength of the P_G1_-family in glucose-limiting conditions [25], the P_G1-3_ variant (described as P_*GTH1*_*‐D1240* in Prielhofer et al. [26]) emerged as the most promising candidate. As a pharmaceutically relevant, stable, and generally well-expressed model protein, a camelid bivalent protein domain antibody (VHH) was chosen [27, 28]. The respective VHH-expressing strain was created previously and assessed in glucose-based small-scale screenings and lab-scale bioreactor-fed-batch cultivations [25].

**Fig. 1:**
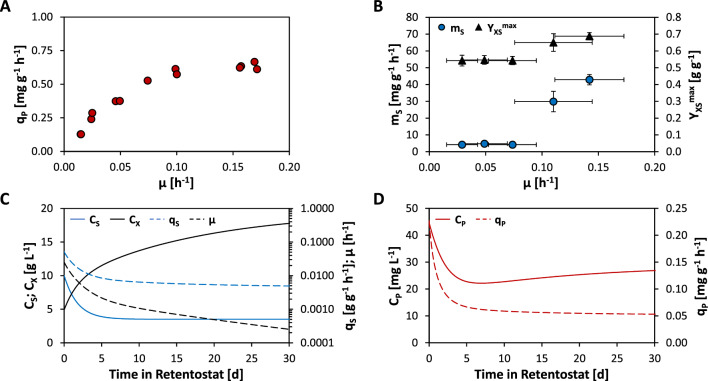
VHH production and growth parameters estimated from chemostat cultivations and corresponding model-based predictions for retentostat cultures. Aerobic, glucose-limited chemostats for parameter estimation were operated at 7 different dilution rate setpoints (*D* = μ) ranging from 0.015 to 0.170 h^−1^. Shown are **A** the biomass-specific VHH secretion rate (q_P_) and **B** the maintenance energy requirement (m_S_) as well as the maximum theoretical biomass yield (Y_XS_^max^) in relation to μ. The dynamics of m_S_ and Y_XS_^max^ were estimated by linear regression analysis on moving windows of the specific glucose uptake rate (q_S_) as determined from three different consecutive dilution rates and at least two individual cultivations per dilution rate setpoint. **C** Model-based predictions for retentostat cultivations of the biomass built-up (Cx), the glucose concentration in the feed (C_S_), µ, q_S_, as well as **D** the VHH concentration in the supernatant (C_P_) and q_P_ were calculated based on the average m_S_ and Y_XS_^max^ values returned from the three sets of q_S_-vs-μ relations for μ ≤ 0.100 h^−1^ from (**B**)

For accurate growth predictions in retentostat cultures, precise estimates of m_S_ and the maximum biomass yield (Y_XS_^max^) of a specific strain are necessary [23]. While m_S_ and Y_XS_^max^ of non-producing *K. phaffii* in glucose-limited retentostat cultures operated at 25 °C were previously determined at 3.1 mg g^−1^ h^−1^ and 0.584 g g^−1^, respectively, evidence suggests that recombinant protein production might lead to a higher energy demand for maintenance [10, 29]. Additionally, earlier fed-batch cultivations demonstrated that raising the cultivation temperature from 25 to 30 °C increased the P_G1-3_-driven production of VHH substantially. As a result, a higher cultivation temperature would likely also result in a higher productivity in the present retentostat study. However, increasing the temperature setpoint adds further uncertainty to the previous estimates of m_S_ and Y_XS_^max^. Hence, m_S_ and Y_XS_^max^ were determined for the VHH-producing strain at 30 °C by carrying out eleven separate aerobic glucose-limited chemostat cultivations that were each run at up to two distinct dilution rates (*D*), ranging from 0.015 to 0.17 h^−1^ (Fig. 1A). This simultaneously made it possible to determine the q_P_-vs-µ dynamics of P_G1-3_ in the *Δflo8* background. In these chemostat cultures, the highest q_P_ of 0.64 mg g^−1^ h^−1^ was measured at the fastest *D*-setpoint of 0.17 h^−1^ (Fig. 1A).

### Model-based predictions of growth and recombinant protein production in retentostat cultivation

According to the Pirt equation (Eq. 1) [30, 31], the energy-limiting substrate consumed in chemostat cultures (q_S_) is distributed over growth, expressed as (μ/Y_XS_^max^), maintenance (m_S_), and, if present, product formation (q_P_/Y_PS_^max^).

1 $${q}_{S}=\frac{\mu }{{Y}_{XS}^{max}}+{m}_{S}+\frac{{q}_{P}}{{Y}_{PS}^{max}}$$

For organisms that exhibit a growth-independent m_S_, this parameter as well as Y_XS_^max^ can be estimated by plotting q_S_ against μ. The inverse of the slope will give Y_XS_^max^, while the intercept corresponds to the maintenance coefficient [19]. However, for a producing organism, q_P_ at the respective µ as well as the maximum theoretical product yield (Y_PS_^max^) must be known to solve the equation. Y_PS_^max^ of VHH on glucose was estimated by employing the *K. phaffii* metabolic model [32]. When assuming that glucose is exclusively spent on maintenance as well as recombinant protein synthesis (but not growth), a Y_PS_^max^ of 0.609 g g^−1^ was predicted. A growth-rate dependency of m_S_ was observed in the previous retentostat experiment. The non-linear relationship of m_S_ and Y_XS_^max^ to μ was identified via sliding-window linear regression analysis on sets of overlapping q_S_-vs-μ relations over defined ranges of consecutive μ [21]. A similar analysis of the current data set demonstrated that m_S_ dropped nearly tenfold between μ of 0.17 and 0.10 h^−1^, but remained stable at even slower growth rates (Fig. 1B). Similarly, Y_XS_^max^ dropped by approx. 30%. Interestingly, the decrease in m_S_ and Y_XS_^max^ of recombinant protein-producing CBS2612 *Δflo8* at 30 °C seems to take place at a higher μ (above 0.10 h^−1^) compared to observations of non-producing CBS7435 *Δflo8* at 25 °C, where substantial changes in m_S_ and Y_XS_^max^ were observed down to a μ of 0.05 h^−1^. Finally, retentostat growth predictions were made using estimates of m_S_ (4.37 ± 0.27 mg g^−1^ h^−1^) and Y_XS_^max^ (0.544 ± 0.002 g g^−1^), which were calculated by averaging the values returned from linear regression analysis from the three sets of q_S_-vs-μ relations of μ ≤ 0.100 h^−1^, where both parameters had stabilized (Fig. 1B). Figure 1C illustrates the predicted profiles of biomass accumulation, µ, and q_S_ for aerobic glucose-limited retentostat cultures based on an optimized feeding strategy determined by model-aided simulation as described in Rebnegger et al. [21] and Vos et al. [23]. Recombinant protein production was also taken into consideration (Fig. 1D). For simplicity, a linear relationship between μ and q_P_ was assumed instead of a higher-order function. It was based on linear regression analysis of the q_P_-vs-μ relationships of the three lowest μ-setpoints of the chemostat cultivation (Fig. 1A), yielding an intercept of 0.051 mg g^−1^ h^−1^, theoretically representing q_P_ at zero growth.

### Recombinant protein synthesis and secretion remain active at near-zero growth rates

**Fig. 2:**
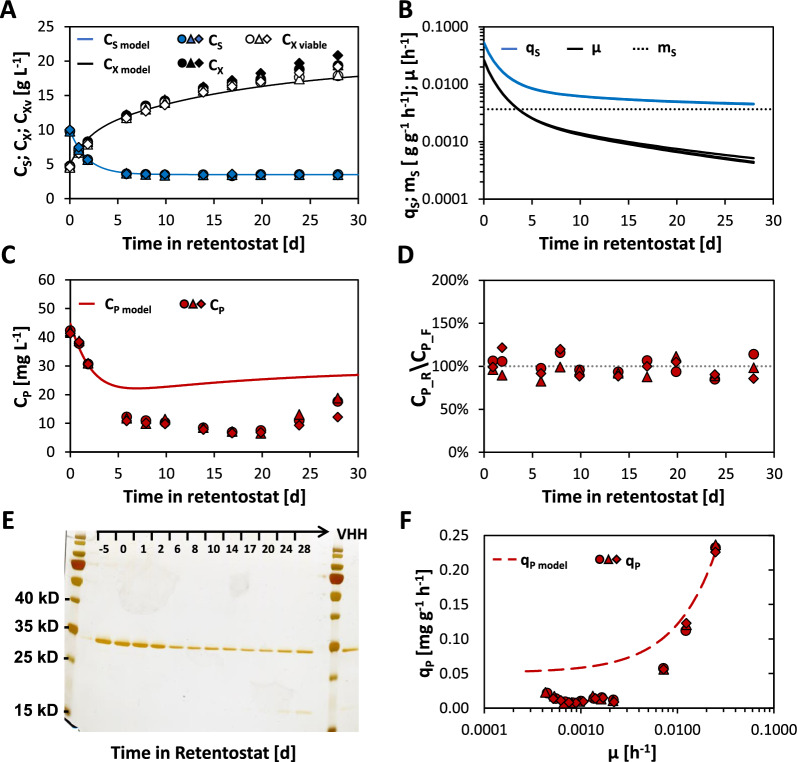
Aerobic, glucose-limited retentostat cultures of VHH-secreting *K. phaffii*. Retentostat cultures were initiated from chemostat cultures operated at *D* = µ = 0.025 h^−1^ at time-point zero. Shown are **A** the total (C_X_) and viable (C_Xv_) biomass accumulation profiles and the glucose concentration (C_S_) in the feed throughout the retentostat phase as well as corresponding model-based predictions. **B** Estimates of the specific glucose uptake rate (q_S_), μ and the average m_S_ calculated based on non-linear regression analysis. **C** VHH titer (C_P_) in supernatants as well as corresponding model predictions based on extrapolation from chemostat data. **D** Ratio of the VHH-titer measured in supernatants (C_P_R_) and filtrates (C_P_F_) at the respective sampling point. **E** Representative SDS-PAGE of retentostat supernatants and VHH standard. Protein bands were visualized by silver-staining. **F** Relationship between q_P_ and µ as well as corresponding model predictions based on extrapolation from chemostat data

Retentostat cultivations were initiated from chemostat cultures operated at *D* = µ = 0.025 h^−1^ and operated for 28 days. Cell viability remained above 90% during the retentostat phase, and the profiles of viable biomass accumulation closely matched those predicted by the model (Fig. 2A). Non-linear regression analysis of biomass accumulation profiles (accounting for recombinant protein production; see Materials and Methods and data presented below as well as Additional file 1: Fig. S1) revealed that on average, the cultures reached a µ of 0.00047 h^−1^ (Fig. 2B), which corresponds to a doubling time of 62 days. The average m_S_ determined from these cultures was 3.7 ± 0.1 mg g^−1^ h^−1^, which is almost 20% lower than the estimate obtained from chemostat cultures utilized for model predictions.

Adaptations to near-zero growth rates were dependent on the length of the preceding chemostat phase (Additional file 1: Table S1 and Fig. S2). In fact, retentostat cultures that were in chemostat phase for a longer period of time (18 days; 10.8 volume changes (VC)) had significantly lower viability and biomass yield as well as a nearly two-fold higher maintenance energy requirement than those cultures whose chemostat phase was restricted to 12 days (7.2 VC). As a result, it was decided to limit the time spent in the chemostat phase prior to the initiation of the retentostat phase accordingly. However, while this phenomenon was not explored further in this study, elucidating the mechanism underlying this behavior might aid in understanding how maintenance is controlled in *K. phaffii* in the future.

This study's main goals included determining if recombinant protein secretion in *K. phaffii* is possible under conditions of almost zero growth. To assess secretion rates and product quality, supernatants and filtrate samples from chemostat and retentostat cultures were analyzed by microfluidic electrophoresis and SDS-PAGE (Fig. 2C–E). Titers were in good agreement with the projected profile during the first two days of retentostat cultivation but remained in the subsequent 8 days approx. twofold and later approx. 3.5-fold below the prediction (Fig. 2C). Interestingly, product concentrations started to rise again between days 20 and 24, and this trend continued until the end of the retentostat. Product concentrations in the filtrate were comparable to those seen in culture supernatants throughout the whole process (Fig. 2D), indicating that minimal product retention in the bioreactor (if any) takes place. Thus, it can be assumed that the observed VHH levels reflect de novo synthesized and secreted product. Furthermore, despite rising biomass concentrations and slightly declining viability, the concentration of host cell protein in the supernatant remained very low. In agreement, a succinylated-casein-based protease assay showed that protease activity in the culture supernatant stayed at background levels until day 20 and slightly increased afterward (Additional file 1: Fig. S3). At the same timepoint, a faint band of approximately 20 kDa occurred (Fig. 2E), which was confirmed by mass spectrometry as a VHH-degradation product (data not shown), suggesting some proteolytic activity at the very end of the retentostat phase.

In the retentostat phase, the measured product titer at a particular time point reflects the biomass concentration, q_P_, and initial titers, as well as wash-out dynamics. To estimate q_P_ in the retentostat phase, a product mass balance was used (see Materials and Methods). Figure 2F shows q_P_ as a function of µ. In line with expectations, q_P_ decreased sharply from 0.23 to 0.06 mg g^−1^ h^−1^ over the first two days of retentostat culturing, while µ dropped from 0.025 h^−1^ to 0.008 h^−1^. At µ below 0.0025 h^−1^, q_P_ stabilized, although at roughly fivefold lower levels than those inferred from chemostat data. Consistent with increasing titers, a significant increase in q_P_ was observed towards the end of the retentostat phase. A drop in P_G1-3_-activity and the resulting lower levels of VHH transcripts during the retentostat phase might explain the significant decline in q_P_. P_G1-3_ was chosen as a promoter for VHH expression as *GTH1* was one of the highest expressed genes (according to previous microarray analysis) throughout glucose-limited retentostat cultures of *K. phaffii* CBS7435 *Δflo8* [21]. Stable integration of the VHH expression cassette throughout the retentostat phase was verified by quantitative PCR (qPCR; Additional file 1: Fig. S4), excluding the possibility that the reduced productivity was due to a decrease in the VHH copy number. To assess recombinant gene expression throughout the retentostat phase and compare it to other genes that show natively high expression levels, qPCR employing standards for absolute transcript quantification was conducted. In agreement with the observed q_P_ profiles (and the obtained RNA-Seq data described below), VHH transcript levels decreased substantially in the early and mid-retentostat phases before returning to chemostat levels at the very end of the retentostat cultivation (Additional file 1: Fig. S5A). *GTH1* expression levels followed a similar pattern but remained at a lower level at the slowest µ. However, absolute *GTH1* levels were slightly higher than those of VHH for most of the investigated sampling points (Additional file 1: Fig. S5B). In the following, VHH transcript levels were also compared to those of *SPI1*, which is one of the strongest, constitutively expressed genes known in *K. phaffii* [33, 34] and ranked among the highest expressed genes in the present RNA-Seq data as well. Indeed, all analyzed samples showed similar or higher VHH levels than those observed for *SPI1*, suggesting that the choice of promoter for retentostat cultivations was indeed appropriate.

### *K. phaffii* changes its biomass composition when approaching near-zero growth rates

**Fig. 3:**
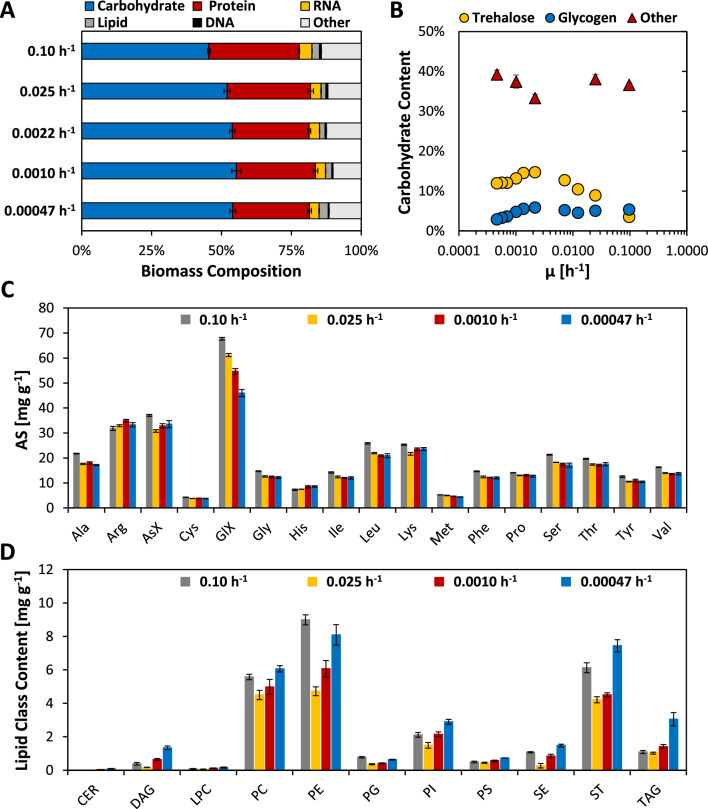
Biomass composition of recombinant *K. phaffii* over a wide range of µ. Samples were taken from separate chemostat cultures operated at *D* = μ = 0.1 h^−1^, retentostat cultures in the chemostat phase (*D* = µ = 0.025 h^−1^; day 0), and throughout the retentostat phase (μ < 0.025 h^−1^). **A** Macromolecular biomass composition; and **B** percentage of storage carbohydrate content and other carbohydrates. **C** Amino acid content measured in hydrolysates of whole cells. GlX represents the sum of Glu and Gln and AsX the sum of Asp and Asn. Tyrosine was not measured. **D** Lipid class content, including ceramide (CER), diacylglycerol (DAG), lyso-phosphatidylcholine (LPC), phosphatidylcholine (PC), phosphatidylethanolamine (PE), phosphatidylglycerol (PG), phosphatidylinositol (PI), phosphatidylserine (PS), sterol ester (SE), sterol (ST), triacylglycerol (TAG). All biomass composition data for the main sampling points is also provided in Additional file 2

Biomass composition has been demonstrated to change with growth rate as well as the respective (limiting) nutrient source in chemostat and retentostat cultures [32, 35–37]. Hence, to investigate to what degree and in what manner the biomass composition changes when recombinant *K. phaffii* approaches near-zero growth rates, samples from separate chemostats operated at *D* = µ = 0.10 h^−1^, cultures in chemostat phase (*D* = µ = 0.025 h^−1^; day 0) and retentostat phase from days 14 and 28 (corresponding to µ of 0.0010 and 0.00047 h^−1^, respectively) were analyzed for their total carbohydrate, protein, RNA, DNA, and lipid content, as well as their amino acid, lipid class, and elemental composition (Fig. 3A, Additional file 1: Tables S2 and S3 as well as Additional file 2). Additionally, a subset of the analyses was also done on retentostat samples taken on day 6 (corresponding to a µ of 0.0022 h^−1^).

Across all growth rates, the largest fraction of biomass was composed of carbohydrates, followed by protein and RNA. In agreement with previous reports of *K. phaffii* X-33 and *S. cerevisiae* grown on glucose, a negative relationship between μ and the total carbohydrate content was observed [32, 35]. However, in the present study, significant changes (*p*-value < 0.05; Additional file 1: Table S4) were only seen between μ of 0.10 and 0.025 h^−1^, while the total carbohydrate content remained rather stable at 54–55% throughout the retentostat phase. Down to a μ of 0.0022 h^−1^, the increase in total carbohydrate content was accompanied by steadily increasing levels of the storage carbohydrate trehalose, which reached a maximum of 14.7% (w/w) at day 6 of retentostat cultivation but started to decrease again thereafter (Fig. 3B). On the other hand, levels of the second main storage carbohydrate, glycogen, remained relatively stable at approx. 5% down to a μ of 0.0014 h^−1^, before decreasing to 2.9% throughout the remaining retentostat phase. Hence, storage carbohydrate metabolism appeared to be regulated similarly in recombinant protein-secreting CBS2612 *Δflo8* as compared to non-producing CBS7435 *Δflo8* [21]. Interestingly, the amount of other carbohydrates also decreased with decreasing μ to 33.3% (w/w) on day 6 before increasing again to 39.2% at the end of the retentostat culture (Fig. 3B).

The amount of total RNA and DNA decreased with decreasing μ (Fig. 3A and Additional file 1: Table S2). The RNA content dropped from 4.6 to 3.8% between μ of 0.10 and 0.025 h^−1^ and reached 3.4% at the end of the retentostat phase, while the total DNA content dropped the most in the early retentostat phase from 0.7 to 0.4%. The total protein content also showed a clear correlation with growth between µ of 0.1 and 0.0022 h^−1^, where it dropped from 32.3 to 27.5%, while it remained relatively stable from the mid-retentostat phase on when cells approached near-zero growth conditions. This pattern was well reflected by the elemental nitrogen content (Additional file 1: Table S3) as well as the amino acid levels, which represented the amino acid content of whole cell hydrolysates (free amino acids are neglectable) (Fig. 3C). Pronounced changes in terms of amino acid level were observed for GlX (referring to the sum of glutamine and glutamic acid), which decreased by approximately 30%. For *S. cerevisiae*, free glutamine has been proposed as a “signature metabolite” that indicates carbon source abundance [11], while glutamic acid pools showed a more diverse pattern. Both free metabolites show a high abundance in comparison to the majority of other free amino acids. It would be tempting to speculate that assumedly low pools of free glutamine at severe calorie restriction might also be reflected in the whole cell hydrolysate glutamine content. Unfortunately, with the method employed in this study, it was impossible to distinguish between glutamine and glutamatic acid.

### Lipidomics reveals an increase in neutral lipid content toward zero growth

The total lipid content and composition of yeasts can be considerably impacted by changes in growth conditions, such as different growth phases, carbon sources, or oxygen availability [38–41]. For *K. phaffii,* significant differences between glucose and methanol conditions were seen regarding triacylglycerol (TAG) content, while over the course of a glycerol batch-phase and a methanol feed-phase, major changes in various phospholipid levels were observed [39, 41]. However, adaptations of the cellular lipidome in relation to growth rate and/or severe calorie restriction have not been investigated so far. Lipidome analysis was done by employing reversed phase liquid chromatography (RP-LC) coupled to high-resolution mass spectrometry (HRMS). Detailed information on the respective lipid class levels as well as the respective species composition is provided in Additional file 2. Overall, the total lipid content showed a more complex pattern compared to other macromolecules, as levels decreased from 2.7 to 1.7% between μ of 0.10 and 0.025 h^−1^, and rose again to 3.2% over the course of the retentostat cultivation. While nearly all individual lipid classes showed a similar trend, phosphatidylethanolamines (PE), sterols (ST), and TAG contributed the most to the total increase in lipid content at the end of the retentostat phase (Fig. 3D). The main sterol in fungi and yeast is ergosterol, which is also the major lipid regulating membrane fluidity and other important biological processes such as endocytosis, cytoskeleton organization, and mating [42, 43]. Furthermore, it is suspected that ST content (and therefore membrane properties) affects recombinant protein secretion [40, 44]. TAG, as well as sterol esters (SE), are neutral lipids that can form lipid droplets (LD), which are very dynamic organelles that are involved in energy and lipid homeostasis, as well as several other biological processes [45, 46]. The size of LDs is reported to increase in late log- and early stationary-phase cultures [47]. SE content in this study followed the general lipid trend, with levels strongly decreasing between µ of 0.10 h^−1^ and 0.025 h^−1^, and gradually increasing again in the retentostat phase. TAG, which has been identified as the primary component of LDs in *K. phaffii* [48], remains at similar levels in the investigated chemostat cultures and up to the mid-retentostat phase, while its levels more than double at the end of the retentostat cultivation. Accordingly, levels of diacylglycerol (DAG), which functions as a precursor for TAG synthesis in the context of LD formation [49], increase nearly eightfold throughout the retentostat phase. Staining of cells with the lipophilic dye Nile red (NR) and subsequent analysis by cytometry and fluorescence microscopy revealed that the fraction of NR-positive cells decreases substantially with decreasing μ (Additional file 1: Fig. S6A), with the exception of the sample taken at the end of the retentostat, for which an increase in NR-staining was observed. However, while the fraction of NR-positive cells in the retentostat phase was much lower, their fluorescence intensity increased more than fivefold (Additional file 1: Fig. S6A). Fluorescence microscopy confirmed these patterns (Additional file 1: Fig. S6B). Remarkably, staining patterns in faster-growing cells were more diffuse, rarely showing a clear LD pattern, while in the retentostat phase, NR-positive cells mostly showed the characteristic LD pattern.

### Global transcriptional regulation patterns of recombinant *K. phaffii* at extremely low growth reveal that growth rate rather than protein production is the major determinant

**Fig. 4:**
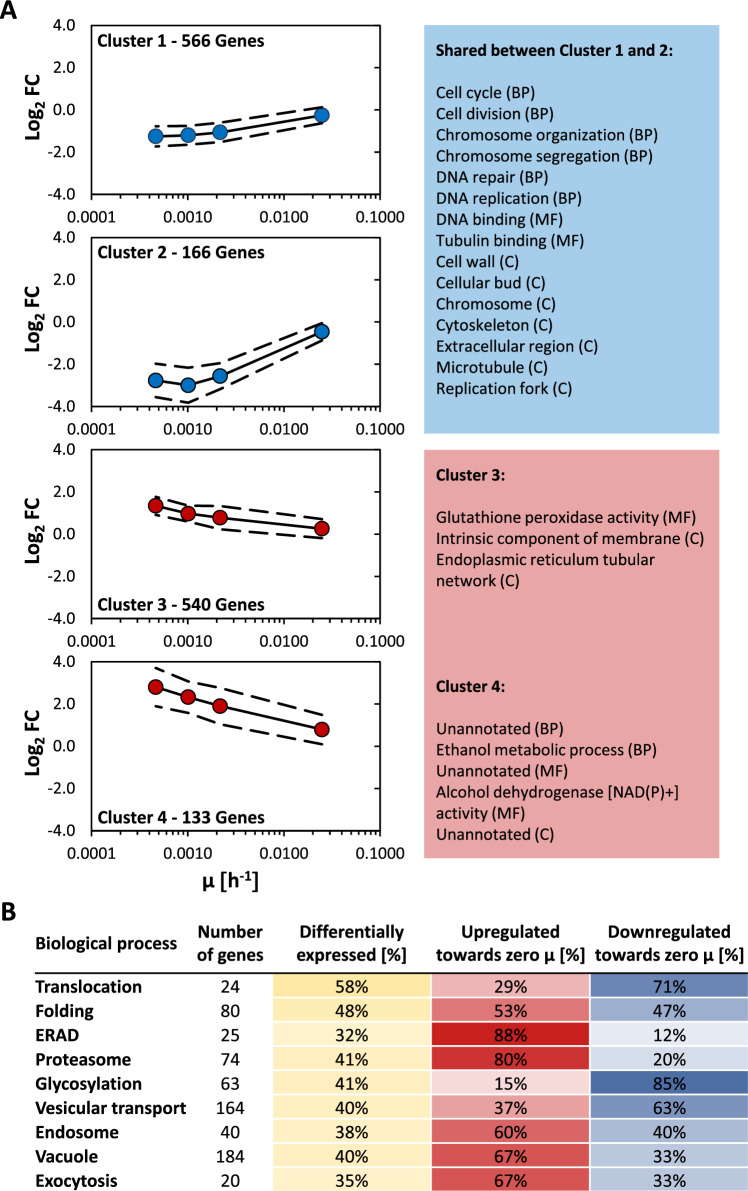
*k*-means cluster analysis, corresponding enriched GO terms, and regulatory trends of secretory pathway genes. **A** Samples for RNA-Seq analysis were taken from separate chemostat cultures operated at *D* = μ = 0.1 h^−1^, retentostat cultures in the chemostat phase (*D* = µ = 0.025 h^−1^; day 0), and throughout the retentostat phase (μ < 0.025 h^−1^; days 6, 14 and 28). Genes that were differentially expressed compared to the highest μ of 0.10 h^−1^ (FC ≥ 2; adj. *p*-value ≤ 0.01) in at least one comparison were grouped into four clusters by *k*-means clustering and analyzed for enriched GO terms. A comprehensive list of all enriched GO terms of the categories biological process (BP), molecular function (MF), and cellular component (CC) identified in the analysis is provided in Additional file 2. Enriched GO terms for Clusters 1 and 2 overlapped to a high degree. The main terms shared between these two clusters as well as all enriched GO terms from Clusters 3 and 4 were listed, respectively. **B** Total numbers of genes allocated to biological processes related to the secretory pathway and relative numbers of regulated genes. Color intensities reflect the degree of regulation of the respective group

Global transcriptional adaptation of VHH-secreting CBS2612 *Δflo8* towards near-zero growth rates was investigated by RNA-Seq. Samples for RNA-Seq analysis were taken from separate chemostats operated at D = µ = 0.10 h^−1^, retentostat cultures operated in chemostat mode just before entering the retentostat phase (*D* = µ = 0.025 h^−1^; day 0) and retentostat samples from days 6, 14, and 28, corresponding to specific μ of 0.0022 h^−1^, 0.0010 h^−1^, and of 0.00047 h^−1^, respectively. Out of 5343 ORFs annotated as protein-encoding genes, 1405 were differentially expressed (FC ≥ 2; adj. *p*-value ≤ 0.01) in at least one comparison against the 0.1 h^−1^ setpoint. *k*-means cluster analysis revealed four primary regulatory patterns (Fig. 4A), with genes displaying either moderate or strong up- or down-regulation toward zero growth. Detailed information on the clusters and corresponding enriched GO terms are provided in Additional file 2. A large majority of the differentially expressed genes (approximately 80%) displayed a rather moderate regulation trend, whereas the distribution between up- (48%) and down-regulated (52%) genes was fairly even.

Significantly enriched GO terms among moderately and strongly downregulated genes towards zero growth (Cluster 1 and 2, respectively) largely overlapped and were almost exclusively related to the (mitotic) cell cycle and respective cell-cycle associated processes, which were also down-regulated with μ in the previous retentostat study using non-producing CBS7435 *Δflo8* [21]. Genes associated with the “cell wall” were also enriched in both clusters, including the major *K. phaffii* cell wall genes *TIP1*, *PIR1, PIR2*, and *BGL2*. Furthermore, nearly all histone-encoding genes were strongly downregulated with decreasing μ and therefore clustered into Cluster 2. Expression of histones has been demonstrated to be regulated in a cell-cycle-dependent manner in *S. cerevisiae* before [50], and strong downregulation of histone genes was observed in previous retentostat cultivations as well [21]. Even though transcriptional downregulation of genes involved in protein biosynthesis is commonly observed in yeast and bacteria in response to slow growth conditions [19], no GO terms related to protein biosynthesis were enriched in Clusters 1 and 2. Examining the genes that encode the 60S and 40S ribosomal subunit proteins in more detail revealed that, in fact, only 22 of the 87 known genes were significantly downregulated in at least one comparison and that the majority of the corresponding transcript levels only exhibited a negative correlation trend towards zero growth. On the other hand, total RNA levels showed a slight but significant drop between μ of 0.10 and 0.025 h^−1^, where only a single ribosomal gene was differentially expressed, indicating that total RNA content and ribosomal gene expression are not strictly correlated in *K. phaffii* under the investigated growth conditions. Mitochondrial translation appears to be even less affected by slow growth, as only one out of 76 genes encoding subunits of the mitochondrial ribosome was significantly downregulated.

In the previous retentostat study of the non-producing *K. phaffii* strain, ergosterol biosynthesis genes were overrepresented among those downregulated towards near-zero growth rates [21]. Despite this not being the case in the current GO analysis, 11 out of 26 genes with a central role in the ergosterol pathway had significantly reduced expression levels in retentostat cultures, and 7 additional genes had a similar regulation trend. Total ST content significantly decreased between μ of 0.1 h^−1^ and 0.025 h^−1^ from 6.1 to 4.2 mg g^−1^, remaining at this level throughout the early retentostat phase, and significantly increased again during the late retentostat phase to 7.4 mg g^−1^. Interestingly, and somewhat in contrast to the lipid content measurements, substantial downregulation of the ergosterol pathway genes was only observed in the sample taken in the early retentostat phase (day 6; μ = 0.0022 h^−1^), but not at the 0.025 h^−1^ setpoint. Furthermore, although ergosterol content at the end of the retentostat phase exceeded those levels measured at the 0.1 h^−1^ setpoint, the expression of most genes remained at those levels observed in the mid-retentostat phase. The transcript levels of *ARE2*, the primary gene responsible for sterol esterification in *K. phaffii* [51], were markedly increased in the early and mid-retentostat phases, which is consistent with rising SE levels in progressing retentostats. The expression profiles of the respective genes involved in TAG biosynthesis, however, did not match its measured content. *LRO1*, the major gene responsible for TAG synthesis in *K. phaffii* [51], was significantly downregulated throughout the retentostat phase, while TAG content remained relatively stable until the second half of the retentostat phase, during which it increased by more than twofold (Fig. 3D). *DGA1*, on the other hand, showed a strong positive regulation trend with a declining growth rate despite being predicted to have a reduced function in TAG synthesis in this yeast under conventional growth conditions. Furthermore, whereas *K. phaffii's* overall PE content varied significantly, neither the expression levels of the two phosphatidylserine decarboxylases, which synthesize PE from phosphatidylserine (PS) (*PSD1* and *PSD2*; [52], nor the levels of the genes that synthesize PE from ethanolamine (*CKI1*, *ECT1*, and *EPT1*) were differentially regulated. In conclusion, the observed changes in lipid class content correlated only poorly with the transcriptional patterns of the genes involved in their production. In Additional file 2, a comprehensive list of lipid biosynthetic genes and the respective differential gene expression data is included.

Only a few enriched GO terms were identified in the two gene sets upregulated towards zero growth. Overrepresented genes that were grouped into Cluster 3 (moderate upregulation) were involved in”glutathione peroxidase activity”, “intrinsic component of membrane” and “endoplasmic reticulum tubular network”. In Cluster 4 (strong upregulation towards zero growth), “unannotated” genes as well as genes with alcohol or aldehyde dehydrogenase activity were found to be overrepresented.

However, while only a limited number of GO terms were found to be enriched, many of the genes that were grouped into clusters 3 and 4 showed the same trend in regulation in the previous retentostat study. For example, genes encoding main stress response transcriptional regulators such as *HAC1*, *MSN4*, *SKO1*, and *YAP1*, respective (oxidative) stress-responsive genes (e.g., *SOD1*, *MXR1-2*, *MXR2-1*, *MXR2-2*, *GRX2*, *HSP31*, *HSP60*), as well as genes involved in transcriptional regulation of nutrient response (e.g. *CAT8-2*, *MIT1*, *GAT1*, and *NRG1*) grouped into clusters 3 and 4. In agreement, many genes with a role in nitrogen catabolite repression (NCR) and alternative carbon source utilization were upregulated as well, including *GAT1* (a main transcriptional activator of NCR) and the main methanol (*AOX1*, *AOX2*, *DAS1*, and *DAS2*) and ethanol (*ADH2*, *ADH600*, and *ADH900*) utilization genes. As described previously, upregulation of NCR genes is not only restricted to low nitrogen conditions but was also observed upon glucose limitation in *S. cerevisiae* [53], and de-repression of methanol and ethanol utilization genes caused by very low glucose levels likely primes the cells to readily metabolize any suitable alternative carbon source if it suddenly becomes available. This behavior represents a “be-prepared” survival strategy and is a common reaction in response to severe calorie restriction in filamentous fungi, yeast, and bacteria [19, 54].

### Transcriptional regulation of secretory pathway genes at near-zero growth rates

Based on the focus of this work on recombinant protein secretion dynamics at extremely slow growth rates, a more in-depth analysis was applied to genes with a function in the secretory pathway, including protein translocation into the endoplasmic reticulum (ER), folding, N- and O-glycosylation, ER-associated protein degradation (ERAD), proteasomal genes and genes of the ubiquitin–proteasome system, vesicular transport, endosome, vacuole, as well as exocytosis. For this analysis, a lower fold-change threshold (1.5 instead of 2) was used to determine whether genes were differentially expressed. Figure 4B provides a summary of the regulatory patterns for the key mechanisms involved in protein secretion. The expression of those genes with a role in co- or posttranslational translocation of the nascent aminopeptide chain into the ER was affected the most by variations in μ. In this cohort, the majority of significantly regulated genes had their expression downregulated, approaching zero growth. Similar findings were made previously for HSA-secreting *K. phaffii* SMD1168H cultivated in glucose-limited chemostat cultures at faster growth rates ranging between 0.015 and 0.15 h^−1^ [10]. Expression of genes involved in protein folding was also strongly affected. However, unlike at faster μ, where a clear positive correlation with the growth rate was observed, the distribution between up- and downregulated genes at extremely slow growth was nearly even. In this regard, it is interesting to note that the expression of *HAC1* (main transcriptional regulator of the unfolded protein response; UPR) was significantly upregulated from mid-retentostat phase on, while it showed a positive correlation with growth in the previous study. However, known UPR targets of *HAC1* were found in both groups, including genes with markedly increased (*KAR2*, *ERO1*, *HSP82*) or decreased (*SCJ1*, *SSA1*, *MPD1*) transcript levels at near-zero growth rates. About 41% of genes with a known function related to core oligosaccharide synthesis, or N- or O-glycosylation, were differentially expressed, and 85% of them were found to be downregulated towards near-zero growth. A positive regulation trend with growth was also observed at faster μ, albeit to a lesser extent. Transcript levels of genes with a function in ERAD as well as the ubiquitin–proteasome system and proteasome-subunit-encoding genes were less affected. However, of those genes that were differentially expressed, 88% of ERAD− as well as 80% of proteasome-related genes were upregulated throughout the retentostat phase. The majority of significantly regulated genes related to the endosome and vacuole showed higher expression levels at extremely slow growth as well, and all genes with a primary function in autophagy displayed a similar trend, further demonstrating that activation of proteolysis is a main response at near-zero growth rates.

Interestingly, while a majority of differentially expressed SNAREs and other genes involved in vesicular transport were downregulated at slow growth, genes involved in exocytosis were mostly upregulated. Taken together, the data shows that numerous processes that drive protein maturation and secretion are impacted at near-zero growth rates and that their regulatory patterns can diverge from those seen in chemostats at faster µ. However, the data also clearly demonstrate that many processes at extremely slow growth are regulated in a manner that seems predominantly detrimental to protein secretion.

## Discussion

In this study, the recombinant protein production kinetics of *K. phaffii* CBS2612 *Δflo8* were examined over a broad range of µ in aerobic, glucose-limited chemostat and retentostat cultures. In the latter, doubling times of approximately 2 months were reached, corresponding to a μ of 0.00047 h^−1^. One of the main goals was to determine whether and, if so, to what extent recombinant protein secretion is sustained at such extremely low growth rates. Maximum specific VHH secretion rates driven by the glucose-limit-induced promoter P_G1-3_ in the *Δflo8* background were observed at the fastest investigated *D* = µ of 0.17 h^−1^. De novo synthesis and secretion of VHH were indeed maintained throughout retentostat cultivation, but the lowest q_P_ was roughly fivefold lower than what was anticipated from chemostat cultivations. Recombinant gene transcript levels significantly decreased along with decreasing secretion levels in the retentostat phase, but to a considerably lesser extent than what was shown in terms of q_P_. However, it should be noted that rising VHH transcript levels did, in fact, correspond to rising productivity at the end of the retentostat phase, demonstrating that transcriptional regulation of the recombinant gene might be partially responsible for the diminished production capacity of the cells, but other factors clearly play a role as well.

Translation is one of the key steps involved in recombinant protein production, and it has been shown in several studies that *S. cerevisiae* and bacteria downregulate the energetically costly translational machinery in progressing retentostat cultures [19, 23]. In contrast, in *K. phaffii*, we did not observe enrichment of GO terms related to protein biosynthesis in the significantly down-regulated gene set, neither in the previous retentostat study [21] nor in this study, although many of the respective genes showed a negative regulation trend. Total RNA (which is to a large degree comprised of ribosomal RNA) as well as total protein levels did indeed drop slightly when cells approached zero growth. However, q_P_ decreased much more rapidly than it would be anticipated from the regulation trends of the translational machinery genes and macromolecular biomass composition data.

Multiple studies have concluded that protein folding and maturation are the main bottlenecks in the production process of secretory recombinant proteins. Consequently, many factors that are involved in protein secretion have been targeted in cell engineering approaches, albeit with varying success (reviewed e.g. by Madhavan et al. [55], Raschmanová et al. [56], and Zahrl et al. [57]). The importance of certain steps of the secretion pathway, such as folding and disulfide bond formation, has previously been established. Furthermore, it was demonstrated for *K. phaffii* and *S. cerevisiae* that increased secretion capacities correlate strongly with the increased expression of genes involved in these key processes, such as translocation, glycosylation, and folding. In contrast, proteolytic processes such as ERAD or vacuolar genes show much lower expression levels at fast µ [10, 13]. In the current study, it was demonstrated that the regulation of secretory pathway genes at near-zero growth deviates from trends observed at faster µ. For example, *HAC1*, the main transcriptional activator of UPR in *K. phaffii*, as well as several of its main target genes, are upregulated towards zero growth, while its expression showed a positive correlation to µ in faster-growing chemostat cultures. *HAC1* overexpression has been demonstrated to increase secreted recombinant protein levels in the past [56], and its upregulation together with some of its targets might benefit protein secretion. However, we observed a simultaneous strong upregulation of genes involved in proteolytic processes, including proteasomal and ERAD genes, as well as vacuolar genes and genes related to autophagy. Furthermore, key genes involved in protein translocation into the ER were strongly downregulated, resulting in a regulatory pattern of secretory pathway genes that seems mostly detrimental to efficient protein secretion.

The choice of a suitable promoter has previously been identified as a key factor in successful recombinant protein secretion at near-zero growth rates, with very little secreted product detected from malate-limited retentostat cultivations of the filamentous fungus *A. niger* [20]. However, the two tested *A. niger* promoters were mainly active at µ > 0.001 h^−1^, while P_G1-3_ in this study allowed for high expression of the VHH across the entirety of investigated growth rates.

Full decoupling of growth and recombinant protein production in *K. phaffii* has been realized in a strain lacking the genes for alcohol oxidase, which represent the first step of methanol utilization, and methanol-based induction of the *AOX1* promoter [58]. Specific secretion rates of the same model protein as employed in the present study throughout the methanol-production phase were on average 0.088 mg g^−1^ h^−1^, and therefore ca. fivefold higher than the q_P_ observed at the end of the retentostat cultivation. MeOH uptake rates were 1.6-fold higher than the reported m_S_ on methanol, while in this study glucose uptake at day 28 exceeded the estimated m_S_ by 25%. Furthermore, the actual non-growth production phase in the methanol-based approach only lasted 72 h, and it is unclear for how long the cells would sustain the observed level of productivity. A possible explanation for higher secretion rates in methanol conditions would be that growth on this carbon source is natively accompanied by very high expression of methanol utilization genes such as Aox1 [59], and methanol-grown cultures might therefore generally sustain high translation activity. However, in a recent study, translation activity at intermediate growth rates was comparable between methanol and glucose-limited conditions [60]. Another explanation would be that expression of the recombinant gene remained higher in the P_*AOX1*_-based system, which, however, would be very difficult to compare directly.

The current study provides a comprehensive analysis of how the macromolecular biomass composition of recombinant *K. phaffii* changes when cells transition to near-zero growth conditions. In essence, the observed regulation trends of the total protein, RNA, and carbohydrate content are similar to what has been observed at faster μ, namely a decrease in total RNA, DNA, and protein content while the total carbohydrate content increases with decreasing μ [32]. In contrast to the total protein, RNA, and carbohydrate content, the DNA content changed the most in the retentostat phase. Lipid content and composition, on the other hand, showed a more complex trend in their regulation. Information on how the macromolecular biomass composition changes when yeast cells approach near-zero growth has so far only been obtained to a limited extent in non-energy-limited *S. cerevisiae* retentostat cultures. In these conditions, Liu et al. [37] reported a significant change in the total protein content for both nitrogen- and phosphor-limited cultures when μ was decreased from 0.025 h^−1^ to below 0.002 h^−1^. Total carbohydrate, RNA, DNA, or lipid content were not investigated, but total carbon content increased in both conditions. Interestingly, changes in total protein content in N- and P-limited cultures of *S. cerevisiae* were much larger compared to what was observed for *K. phaffii* in this study.

Coltman et al. [67] used the here presented cultivation and biomass composition data to investigate the metabolic rewiring of *K. phaffii* in response to extremely slow growth rates by flux balance analysis and flux sampling. According to this study, *K. phaffii* mainly adapted to severe calorie restriction by reducing flux through the pentose phosphate pathway, and channeling a greater proportion though glycolysis and the TCA cycle. This contributed, among others, to a higher yield of ATP per consumed glucose.

The amino acid composition of the whole cell lysate changed only slightly with μ. Large changes were only detected for the sum parameter of glutamic acid and glutamine. When comparing the fast μ set-point in this study to the respective amino acid distribution described by Carnicer et al. [61] for 21% oxygen, most values are within good agreement (< 16% relative change, Additional file 1: Table S5). Cysteine and methionine, however, were not protected via oxidation prior to HCl hydrolysis in the previous study, resulting in their underestimation [62]. Consequently, values for methionine were 1.6-fold higher and for cysteine even more than eightfold higher in our study when compared to the highest levels reported for *K. phaffii* by Carnicer et al. [61]. In contrast to Carnicer et al. [61] we do not report any values for tryptophan as it is known to fully degrade during HCl hydrolysis and could also not be detected after MSA hydrolysis. We obtained 15 to 16% higher values for AsX, proline, serine, and tyrosine, all of which were measured after MSA hydrolysis. Serine, tyrosine and threonine are known to be partially degraded during HCl hydrolysis [63], which was the only hydrolysis method employed by Carnicer et al. [61]. On the other hand, we observed 5 to 16% lower values for valine, GlX, alanine, isoleucine, histidine, and arginine. This finding can only in part be explained by the fact that the values being compared here are percentages. Besides the application of two independent acidic hydrolysis methods, another major difference in methodology employed in comparison to previous studies analyzing *K. phaffii* protein amino acid composition on glucose and other carbon sources [32, 61, 64], is the fact that our analytical workflow made use of MS detection. Hence, we could base quantification on external calibration and internal standardization with heavy isotope-labeled (i.e., chemically identical) internal standards. These internal standards were added prior to hydrolysis to improve the accuracy of sample preparation and analysis, including this first sample preparation step. Consequently, the method employed in this study should reflect the actual amino acid composition of hydrolyzed *K. phaffii* whole cells more accurately than the previous measurements employing only HCL hydrolysis [32, 61, 64] and therefore represents a valuable contribution to the biomass characterization of *K. phaffii* in general. It has to be mentioned, however, that isoleucine, valine and leucine might be also underestimated in our study as hydrolysis is not 100% efficient for certain amino acid sequences [62, 63].

Changes in total lipid content as well as lipid class and species composition were analyzed by RP-LC-HRMS. Interestingly, the overall lipid content was approximately 40% lower in cells cultivated at a μ of 0.025 h^−1^ compared to those grown at 0.1 h^−1^ in chemostat cultures, but the trend fully reversed throughout retentostat cultivation. At the very end of the retentostat phase, total lipid levels even exceeded those measured from the 0.1 h^−1^ chemostat samples. This increase was to a large degree based on the rise in PE, ST, and TAG levels. PE and the main sterol in yeast, ergosterol, are polar and major components of cellular membranes, while TAG is a neutral lipid and the main component of lipid droplets in *K. phaffii* [48, 65, 66]. While a wide array of functions are attributed to lipid droplets, their core and potentially most ancient function is to serve as a nutrient reservoir [49]. Accordingly, lipid droplet formation in *S. cerevisiae* is reported to accelerate in the late exponential phase when cells enter the diauxic shift and nutrients become increasingly limiting [47, 49]. Also, in *K. phaffii*, LD formation is increased in late exponential and early stationary phase [48]. In this regard, it is somewhat curious that we see the lowest levels of TAG and sterol ester, the second major neutral lipid present in lipid droplets, in the 0.025 h^−1^ chemostat samples. In fact, lipid droplets were mostly observed later in the retentostat phase. Hence, lipid droplet formation might be regulated differently in cells that are slowly transitioning to near-zero growth conditions, in contrast to those cells that find themselves in much more rapidly depleting nutrient conditions at the end of batch culture.

## Conclusions

This study demonstrates that the production of recombinant secretory proteins by *K. phaffii* is feasible in near-zero growth conditions in glucose-limited retentostat cultures, although titers and production rates remained lower than predicted based on extrapolations from faster μ investigated in chemostat cultures. The comprehensive macromolecular and omics data set pinpoints potential cell engineering targets, such as the translocation process that has recently been identified as rate-limiting [68]. Changes in lipid metabolism reveal a complex adaptation pattern towards very slow growth rates. The higher abundance of storage lipids as well as storage carbohydrates indicates that the cells are preparing for long-term survival. On the other hand, the restored abundance of membrane-forming lipid classes such as PE, PC, and sterols towards zero growth might contribute to the still active secretory pathway under these scarce conditions.

## Materials and methods

### Strains and culture conditions

As a base strain, *K. phaffii* CBS2612 (CBS-KNAW Fungal Biodiversity Centre, Centraalbureau voor Schimmelcultures, Utrecht, The Netherlands) was used. The construction of the respective Δ*flo8* mutant and the VHH-secreting strain has been described elsewhere [21, 25]. Strain propagation was routinely done on YPD agar plates at 30 °C for 48 h. As precultures for bioreactor cultivations, 100 mL of YPD liquid media in a 1 L shake flask were inoculated with a cryo vial of the respective strain and incubated overnight at 25 °C and 180 rpm on a rotary shaker. YPD solid and liquid media contained per liter 20.0 g of soy peptone, 10.0 g of yeast extract, 20 g of glucose, and, in the case of solid media, 20 g of agar–agar. If appropriate, Zeocin was added to the media at 50 μg L^−1^.

### Bioreactor cultivations

Aerobic, glucose-limited, chemostat cultivations for the determination of the strain-specific growth parameters and q_P_-to-μ ratios were carried out in 1.8 L parallel benchtop bioreactors (Eppendorf, Germany) at 7 distinct dilution rate setpoints, reaching from 0.015 to 0.170 h^−1^. Two dilution rate setpoints were analyzed per individual chemostat cultivation. Samples were taken after seven volume changes had passed at a respective dilution rate. Precultures were grown as described above, harvested, washed, and resuspended in chemostat media, and used to inoculate the prefilled bioreactors to an OD_600_ of 0.2. Chemostat cultures were operated at a working volume of 0.6 L, except for chemostats preceding the retentostat phase as well as an additional set of chemostats carried out at a dilution rate of 0.1 h^−1^, where the working volume was 1.4 L. The working volume was kept steady using a level sensor. Reactors were stirred at a constant rate of 700 rpm and aerated at a constant airflow of 0.5 L min^−1^. The dissolved oxygen (DO) was monitored with a DO probe and controlled at 30% throughout initial batch cultures. Throughout all chemostat and retentostat cultures, DO remained above 50%. Hence, adjusting the level of oxygenation was not necessary to ensure sufficient oxygen supply. The culture temperature was controlled at 30 °C, and the pH was kept at 5.0 by the automated addition of a 12.5% ammonia solution. Chemostat medium contained (per liter) 10 g glucose, 5 g (NH_4_)_2_SO_4_, 3 g KH_2_PO_4_, 0.5 g MgSO_4_·7H_2_O, 1.5 mL of trace metal solution [69], 0.8 mL of 0.1 g L^−1^ biotin solution (Sigma-Aldrich, USA), and 0.25 g Pluronic 6100 PE antifoaming agent (BASF, Germany).

Aerobic, glucose-limited retentostat cultivations were performed in a similar manner as described previously [21]. Briefly, retentostat cultures were initiated from steady-state chemostat cultures operated at *D* = 0.025 h^−1^ by switching the reactor effluent to an outflow port equipped with an autoclavable filter assembly (TRACE Analytics). By this, a perfusion rate of 0.025 h^−1^ was maintained throughout the retentostat phase. The bioreactor system, main culture conditions (aeration, pH, and temperature), as well as the medium composition (except for glucose), were as described above. Retentostats were operated at a working volume of 1.4 L To predict retentostat growth dynamics, the predictive biomass accumulation script of Rebnegger et al. [21] was used. The maintenance energy requirement (m_S_), the maximum theoretical yield (Y_XS_^max^) and a linear function for the specific productivity (q_P_) in relation to the specific growth rate (μ) were deduced from the chemostat data as described below. According to the chosen feeding regime, the working volume of the mixing vessel (a re-designated 1.8 L Eppendorf benchtop bioreactor) was kept steady at 1.5 L by using a level sensor, and the glucose concentration in the media was gradually decreased from 10 to 3.5 g L^−1^ upon initiation of the retentostat phase. A schematic diagram of the retentostat setup is provided as Additional file 1: Fig. S7.

#### Viability measurement

Viability was determined based on propidium iodide staining. Briefly, samples were diluted with PBS to an OD_600_ of 0.5 and stained with propidium iodide stock solution to a final concentration of 2.0 μmol L^−1^. Samples were then analyzed on a Cytoflex S (Beckman Coulter).

#### Biomass measurement

OD_600_ was measured on a lab photometer. Biomass concentration (yeast dry mass, YDM) was routinely determined in technical duplicates. For this purpose, 5 mL of culture broth was harvested by centrifugation at 4000 *g* for 5 min, washed twice with demineralized water, transferred to a pre-weighed beaker, and dried at 105 °C for at least 48 h.

#### Measurement of the recombinant protein

Quantification of the recombinant secreted protein in the supernatant and filtrate was done by microfluidic capillary employing the LabChip GX/GXII System (PerkinElmer). The consumables Protein Express Lab Chip (760499, PerkinElmer) and Protein Express Reagent Kit (CLS960008, PerkinElmer) were used. Chip and sample preparation were done according to the manufacturer's recommendations. Quantification was done by employing the LabChip software provided by the manufacturer. For protein gel analysis of culture supernatants, NuPAGE® Novex® 12% Bis–Tris gels and MOPS buffer were used according to the manufacturer’s instructions. Proteins were visualized by silver staining as described in Heukeshoven and Dernick [70].

*Calculations of m*_*S*_*, Y*_*XS*_^*max*^* and Y*_*PS*_^*max*^* for retentostat growth predictions* To determine how m_S_ and Y_XS_^max^ of the producing strain change with μ, q_S_ was determined at D = μ ranging from 0.015 to 0.170 h^−1^ in chemostat cultures, and regression analysis on moving windows of three q_S_ values calculated at three consecutive μ values was done. This analysis is based on the Pirt equation, where the intercept at the y-axis gives m_S_ and the reciprocal of the slope is Y_XS_^max^ [19, 30]. To fully solve the equation, the maximum theoretical product yield (Y_PS_^max^) needs to be known, which was calculated in COBRApy [71] by employing the *K. phaffii* genome-scale metabolic model, iMT1026v3 [32]. iMT1026v3 was constrained with a non-growth associated maintenance of 2.81 mmol ATP g^−1^ h^−1^ and a glucose uptake rate of 2.46 mmol g^−1^ h^−1^. The oxygen and CO_2_ exchange rates were unbounded. The reactions for the VHH production were introduced into iMT1026v3, accounting for amino acid polymerization and the associated energetic requirements (4.3 mmol ATP per mmol g^−1^ amino acid) according to [72]. Y_PS_^max^ was calculated at 0.609 g g^−1^ by maximizing the secretion reaction for the assembled VHH.

#### Retentostat non-linear regression analysis and estimation of specific productivity

Regression analysis of biomass accumulation in retentostats for the determination of m_S_, q_S_ and μ was achieved by least-squares regression analysis employing a Python implementation of the model described in Rebnegger et al. [21]. A modification was made in the q_P_-μ relationship; rather than assuming a constant linear relationship, a Gompertz equation was fitted to the determined q_P_ values (see below) and the μ values determined from point-wise interpolation of the C_X_ values in the retentostat, in addition to the q_P_ and μ of the separate chemostat cultivations. The fitted function was incorporated into the regression model (the updated model can be found at https://github.com/bcoltman/Kphaffii_NearZero) and used instead of the linear relationship described previously.

A piecewise product balance accounting for production, accumulation and washout was used to calculate q_P_. To achieve this, the biomass (C_X_) and measured product (C_P_M_) concentrations were linearly interpolated between the sampling points at approx. 24-h intervals. The concentration of the product in the supernatant, if productivity was zero between time points (C_P_0_), was calculated via Eq. 2, accounting for wash-out kinetics where φ is the perfusion rate.

2 $${C}_{{P\_0}_{i}} ={C}_{{P\_M}_{i-1}} * {e}^{\left(\left({t}_{i}- {t}_{i-1}\right)* -\varphi \right)}$$

The concentration of de novo secreted product (C_P_N_) within a given time period was calculated according to Eq. 3:

3 $${C}_{{P\_N}_{i}} ={C}_{P{\_M}_{i}}-{C}_{P{\_O}_{i}}$$

And q_P_ was calculated according to Eq. 4:

4 $${q}_{P} =\frac{{C}_{{P\_N}_{i}}}{\left(\frac{{C}_{{X}_{i}}+ {C}_{{X}_{i-1}} }{2}\right)*\left({t}_{i}-{t}_{i-1}\right)}$$

#### HPLC analysis of sugars, organic acids, and alcohols

Mixing vessel samples, culture supernatants, and filtrates were analyzed on a high-performance liquid chromatography (HPLC) setup (Shimadzu Corporation, Japan) equipped with a Rezex ROA-organic acid H + column (300 mm by 7.8 mm; Phenomenex, USA). A refraction index detector (RID-10A; Shimadzu Corporation, Japan) was used for quantitation. The column was operated at 60 °C, the flow rate was set at 1 mL min^−1^, and 4 mM H_2_SO_4_ served as the mobile phase. Before injection, 0.9 mL of sample were mixed with 0.1 mL of 40 mM H_2_SO_4_ and filtered through a 0.20-μm regenerated cellulose (RC) membrane filter. The injection volume was 10 μL.

#### Sample preparation for biomass composition analysis

Samples for biomass analysis were washed with cold deionized water, snap-frozen in liquid nitrogen, and stored at − 70 °C until further use. With the exception of biomass samples for lipidomics, samples were lyophilized before the respective analysis, and the lyophilized biomass was aliquoted into appropriate amounts into sealable glass vials using a microbalance.

*Macromolecular biomass composition analysis Total carbohydrate content* was determined based on the phenol method [73]. Briefly, 0.15 mg of lyophilized biomass were resuspended in 1 mL of deionized water and mixed with 1 mL of a 5% phenol solution. Afterwards, 5 mL of 96% sulfuric acid were added, and the solution was incubated for 45 min at room temperature. Subsequently, the absorbance was measured at 488 nm, using glucose solutions as a standard. Results were corrected for the presence of nucleic acid pentoses using a relative absorbance of 0.445 AU and 0.264 AU for RNA and DNA, respectively.

*Storage carbohydrate content* in the form of glycogen and trehalose was measured as described previously [21], with the exception that for quenching and washing of the pellet, a pre-cooled (− 20 °C) “low salt” (0.05 M NaCl) and “high pH” (0.125 M Tris HCl; pH 8.2) MeOH (60%) quenching solution was used instead of pure methanol [74].

*Total RNA content* was determined according to Benthin et al. [75]. Briefly, 4 mg of lyophilized biomass were resuspended in 1 mL of cold 0.7 M HClO_4_. Subsequently, 950 µL of this suspension was transferred to a 15 mL polypropylene tube and mixed with 2050 µL of cold 0.7 M HClO_4_. Afterwards, the biomass was centrifuged, washed twice with 3 mL of cold 0.7 M HClO_4_ (4200 g for 10 min at 4 °C), and resuspended in 3 mL of 0.3 M KOH to lyse cells. The lysate was incubated at 37 °C for 1 h, and, after cooling, 1 mL of cold 3 M HClO_4_ was added for neutralization, and the sample was centrifuged again. The supernatant was then transferred to a fresh polypropylene tube, and the extraction step was repeated two more times. The respective extracts were pooled and centrifuged to remove KClO_4_ precipitates before the absorbance was measured at 260 nm against a blank. The RNA quantity was calculated by assuming 1 unit of absorbance at 260 nm corresponds to 0.038 mg RNA mL^−1^ and considering sample dilution.

*Total DNA content* was analyzed by adapting the Schmidt-Thannhauser-Schneider method according to Herbert et al. [73]. Briefly, 2.5 mg of lyophilized biomass were treated in the same manner as for total RNA content analysis, with the exception that the whole procedure was downscaled to fit into 1.5 mL screw cap centrifugation tubes. After the RNA extraction was completed, the remaining pellet (containing the DNA) was resuspended in 1 mL of 0.5 M HClO_4_, incubated for 15 min at 70 °C, and then centrifuged for 2 min at 12,000 *g* and 4 °C. The supernatant was collected in a fresh tube, and the DNA extraction step was repeated two more times using 0.25 mL of 0.5 M HClO_4_. The DNA extracts were pooled, and the absorbance was measured at 260 nm against a blank. The DNA quantity was calculated by assuming 1 unit of absorbance at 260 nm corresponds to 0.050 mg DNA mL^−1^ and considering sample dilution.

*The total protein content* was measured by the Biuret method [73]. Briefly, 4 mg of lyophilized biomass were resuspended in 500 μL of deionized water. Samples were then split into two 250-μL aliquots and mixed with 125 μL of 3 M NaOH. Heat extraction was done for 5 min at 99 °C. Subsequently, 125 µL of a 2.5% CuSO_4_ solution was added. Afterwards, the samples were centrifuged for 5 min at 12,000 *g*, and a 200 µL aliquot of the supernatant was transferred to a transparent 96-well plate. The absorbance was measured on a Tecan reader at 488 nm, using BSA solutions as standard.

#### Protein amino acid composition analysis

For amino acid analysis, approximately 3 mg of lyophilized cells were hydrolyzed, both using (1) HCl after prior oxidation of sulfur-containing amino acids and (2) using methanesulfonic acid for the analysis of unstable amino acids. Tryptophan is degraded in both hydrolysis methods and, hence, could not be quantified. As glutamine is converted to glutamate and asparagine to aspartate during hydrolysis, the sums of these pairs were evaluated (GlX and AsX, respectively). For the analysis of most amino acids, an automated sample preparation robot (MPS2, Gerstel) was used for just-in-time online derivatization with N-(tert butyldimethylsilyl)-N-methyltrifluoroacetamide with 1% tert butyldimethylchlorosilane prior to gas chromatography—tandem mass spectrometry measurements on a GC–MS/MS (7890B GC with 7010B triple quadrupole GC–MS, Agilent Technologies) equipped with a 5% diphenyl 95% dimethyl polysiloxane analytical GC column (30 m, 0.25 mm ID, 0.25 mm film thickness, Phenomenex ZB5 MS; detailed column setup in Additional file 1). Two amino acids, namely arginine and histidine, were analyzed via LC–MS/MS on a HILIC column (XBridge Amide analytical column;150 × 2.1 mm, 3.5 µm particle size, Waters, Milford, USA). Data evaluation was based on external calibration and internal standardization (amino acid standards of certified purity and stability were used). The internal standard, a heavy isotope-labeled ”Cell Free” Amino Acid Mix (U-13C, 97–99% & U-15 179 N, 97–99%, Cambridge Isotope Laboratories), was added to the samples prior to hydrolysis. For a detailed description of hydrolysis and GC–MS analysis, see Szeliová et al. [76] and for modifications of this method and the LC–MS/MS method, see Additional file 1.

*Lipidomics * Lipid class quantification followed the protocol of Schoeny et al. [77] applying a sample-matched ^13^C-labeled internal standard (ISTD) [78] on reversed-phase liquid chromatography (RP-LC) coupled with high-resolution mass spectrometry (HRMS). In brief, a homogenous cell mixture of the samples and the ISTD was extracted via an adopted Folch protocol [79]. An Acquity HSS T3 (2.1 mm × 150 mm, 1.8 µm, Waters) equipped with a VanGuard Pre-column (2.1 × 5 mm, 100 Å, 1.8 µm, Waters) was used on a Vanquish™ Horizon HPLC (Thermo Scientific) coupled to a high field Q Exactive HF quadrupole-Orbitrap mass spectrometer (Thermo Scientific). As eluents, acetonitrile (ACN)/H_2_O (3:2, v/v) and IPA/ACN (9:1, v/v), both containing 0.1% formic acid and 10 mM ammonium formate, were used in a 30 min run. Lipid identification was performed with LipidSearch 4.2 from Thermo Scientific, and quantitative data processing was performed in R/R studio. A more detailed description is provided in Additional file 1.

#### RNA isolation and quantitative PCR

RNA was isolated from cell pellets from chemostat and retentostat cultivations using the Tri reagent according to the supplier's instructions (Ambion, USA). Cells were disrupted by glass beads using a ribolyser (MP Biomedicals). To remove residual DNA, the RNA samples were treated with the DNA-free™-kit (Ambion) according to the manufacturer’s manual. Subsequently, RNA quality, purity, and concentration were analyzed by a NanoDrop™ spectrophotometer and Bioanalyzer (Agilent). For cDNA synthesis for qPCR, the Biozym cDNA synthesis kit (Biozym) in combination with oligo(dt)_23_ primers (NEB) was used. Quantitative PCR was carried out using the Biozym Blue Probe qPCR kit on a Rotor Gene Q instrument (Qiagen). Changes in transcript levels were calculated relative to the reference sample after normalization to *ACT1* (PP7435_Chr3-0993) expression using the threshold cycle method of the Rotor Gene software. A more detailed description and a list of the used primers is provided in Additional file 1.

#### RNA-Seq library preparation

Library generation and sequencing were performed at the Vienna Biocenter Core Facility NGS Unit (www.vbcf.ac.at). Ribosomal RNA was depleted from total RNA (NEB) using an in-house method. The resulting ribo-depleted RNA was then fragmented, and cDNA libraries were generated using the NEBNext® Ultra™ II Directional RNA Library Prep Kit for Illumina (NEB). Libraries were sequenced as paired-end 150-base pair reads on an Illumina NovaSeq 6000 using the SP workflow.

#### RNA-Seq analysis

The raw BAM files were merged and sorted using samtools 1.9 [80]. For trimming, cutadapt 1.18 [81] was used. Alignment was performed with BowTie2 2.3.5.1 [82, 83] against the reference sequence of *K. phaffii* CBS7435. As the count quantification with kallisto depends on a transcript index of the reference sequences, this index was created with kallisto index v0.46.0 [84] and the latest *K. phaffii* CBS7435 annotation (FR839628.1, FR839629.1, FR839630.1, FR839631.1, FR839632.1). Accordingly, the count data of all sample reads was calculated with kallisto quant v0.46.0. Differential expression analysis [85] for each sample comparison was performed with several R v4.0.3 packages, which are tximport v1.18.0 [86], readr v1.1.0 [87], and DESeq2 v1.30.0 [85, 88, 89].

#### Cluster and GO enrichment analysis

Differentially expressed genes were grouped according to their expression profiles by the Genesis software tool [90], employing *k*-means clustering. According to figure-of-merit analysis, a *k* value of 4 was determined to be the ideal number of clusters for the data set. For the determination of enriched Gene Ontology (GO) terms in the respective clusters, the online Generic GO Term Finder tool (http://go.princeton.edu/cgi-bin/GOTermFinder) and *Saccharomyces* Genome Database (SGD) annotations were used. The cut-off for the corrected *p*-value (Bonferroni correction) was set to 0.05, and a *K. phaffii*-specific background list comprised of all annotated genes and genes with unknown function was provided.

### Supplementary Information

**Additional file 1:.** Additional Figures and Tables as well as additional Materials and Methods.

**Additional file 2.** Detailed biomass composition measurement data for triplicate chemostat and retentostat cultivations, clusters identified by *k*-means cluster analysis and corresponding enriched GO terms as well as Log_2_ FC data and adjusted *p*-values for *K. phaffii* genes with a role in lipid biosynthesis.

## Data Availability

All data are included in the manuscript or its supplements. The regression model and its associated data and results are available at https://github.com/bcoltman/Kphaffii_NearZero. RNA-Seq raw data was deposited at NCBI SRA with the accession ID PRJNA1013119.
