# Supplementary material for: Protein production dynamics and physiological adaptation of recombinant Komagataella phaffii at near-zero growth rates
Source: Microb Cell Fact. 2024 Feb 8;23:43. doi: 10.1186/s12934-024-02314-3 (PMC10851509; doi:10.1186/s12934-024-02314-3)
Supplement: Supplementary file 1 — Additional file 1:. Additional Figures and Tables as well as additional Materials and Methods. [file 12934_2024_2314_MOESM1_ESM.docx]

**Supplementary Material for Publication**

**Protein production dynamics and physiological adaptation of recombinant *Komagataella phaffii* at near-zero growth rates**

Rebnegger Corinna^1,2,3^, Benjamin L. Coltman^1,2^, Viktoria Kowarz^1,2^, David A. Peña^2^, Axel Mentler^4^, Christina Troyer^5^, Stephan Hann^5^, Harald Schöny^6^, Gunda Koellensperger^6,7^, Diethard Mattanovich^1,2,3^ and Brigitte Gasser^1,2,3. §^

^1^ CD-Laboratory for Growth-decoupled Protein Production in Yeast at Department of Biotechnology, University of Natural Resources and Life Sciences (BOKU), Vienna, Austria

^2^ University of Natural Resources and Life Sciences, Vienna, Department of Biotechnology, Institute of Microbiology and Microbial Biotechnology, Muthgasse 18, 1190 Vienna, Austria

^3^ ACIB GmbH, Muthgasse 11, 1190 Vienna, Austria

^4^ University of Natural Resources and Life Sciences, Vienna, Department of Forest- and Soil Sciences, Institute of Soil Research, Peter-Jordan-Straße 82, 1190, Vienna

^5^ University of Natural Resources and Life Sciences, Vienna, Department of Chemistry, Institute of Analytical Chemistry, Muthgasse 18, 1190 Vienna, Austria

^6^ Department of Analytical Chemistry, Faculty of Chemistry, University of Vienna, Waehringer Straße 38, 1090, Vienna, Austria

^7^ Vienna Metabolomics Center (VIME), University of Vienna, Althanstraße 14, 1090, Vienna, Austria

^§^ corresponding author:

Assoc. Prof. Dr. Brigitte Gasser

Institute of Microbiology and Microbial Biotechnology (IMMB), Department of Biotechnology, University of Natural Resources and Life Sciences (BOKU), Vienna

Muthgasse 18, 1190 Vienna, Austria

Email: [brigitte.gasser@boku.ac.at](mailto:brigitte.gasser@boku.ac.at)

Phone: +43 1 47654 79033

FAX: +43 1 47654 79009

**This File contains:**

**Supplementary Figures S1, S2, S3, S4, S5, S6 and S7.**

**Supplementary Tables S1, S2, S3, S4, S5 and S6.**

**Supplementary Materials and Methods.**


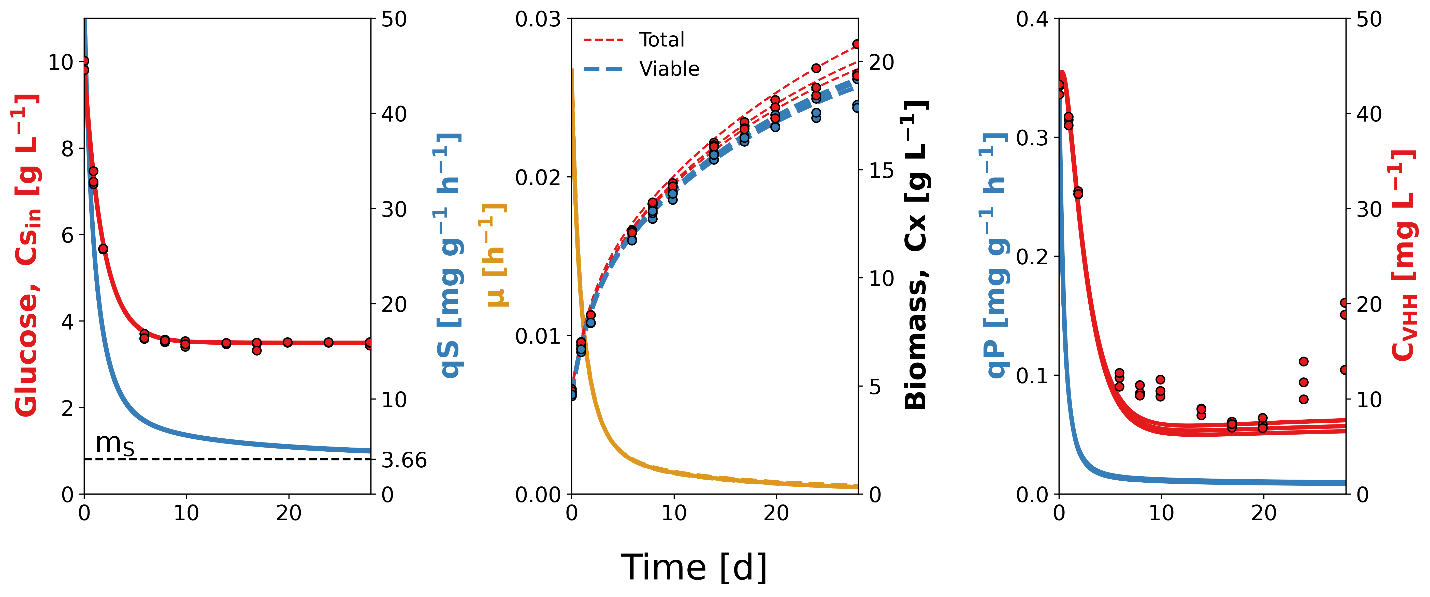


**Figure S1: Non-linear regression analysis of the different retentostat experiments**. Closed circles represent the measured glucose concentration in the feed (C_S_), the total and viable biomass concentration (C_X_) as well as the VHH concentration and the estimated q_P_ values. Lines represent the data from the non-linear regression analysis. The regression model and its associated data and results are available at https://github.com/bcoltman/Kphaffii_NearZero.

**Table S1: Comparison of different culture parameters for retentostat cultures of VHH-secreting *K. phaffii* initiated from chemostat cultures operated for a shorter (SC) or longer (LC) time period.**

|  | **Chemostat Phase** | | | | | | **Retentostat Phase** |
| --- | --- | --- | --- | --- | --- | --- | --- |
| **Cultivation** | ***D* = µ [h^-1^]** | **Time [d]** | **VC** | **Generations** | **C_X_ [g L^-1^]** | **Viability [%]** | **Time [d]** |
| **SC: #1, #2, #3** | 0.0249 ± 0.0001 | 12 | 7.2 | 10.4 | 4.78 ± 0.06 | 95.9 ± 0.7 | 28 |
| **LC: #1, #2, #3** | 0.0248 ± 0.0001 | 18 | 10.8 | 15.6 | 4.80 ± 0.10 | 95.6 ± 0.4 | 28 |

**
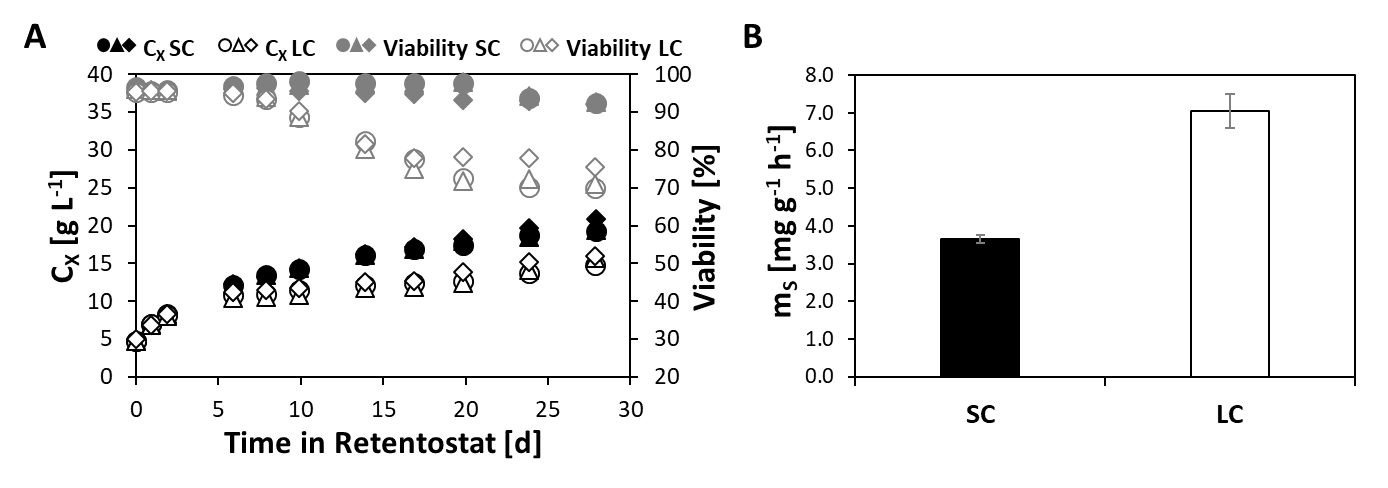
Figure S2.** **Aerobic, glucose-limited retentostat cultures of VHH-secreting *K. phaffii* initiated from short (SC; 7.2 volume changes (VC)) and long (LC; 10.8 VC) chemostat cultures.** Retentostat cultures were initiated from chemostat cultures operated at *D*=µ=0.025 h^-1^ at time-point zero. **A)** Biomass accumulation profiles as well as viability based on propidium iodide staining and cytometry of SC and LC cultures. **B)** The average maintenance energy requirement m_S_ of SC and LC cultures as determined by non-linear regression analysis of viable biomass accumulation profiles in the retentostat phase.

**
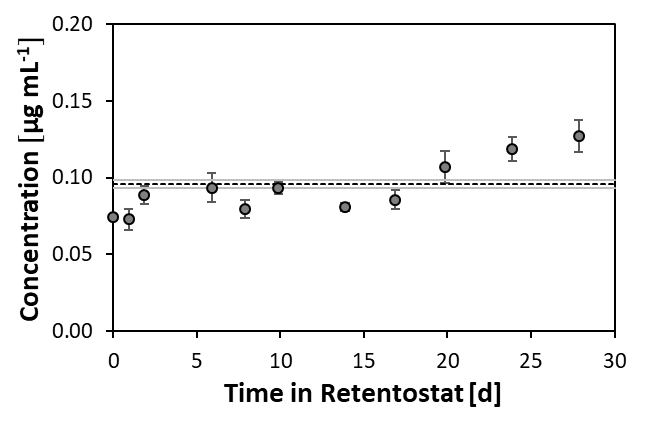
**

**Figure S3. Protease activity in retentostat cultures.** Closed circles represent average values of protease concentrations based on trypsin standards measured in retentostat supernatants at different time points. The dotted line represents the background signal that was obtained with fresh media.


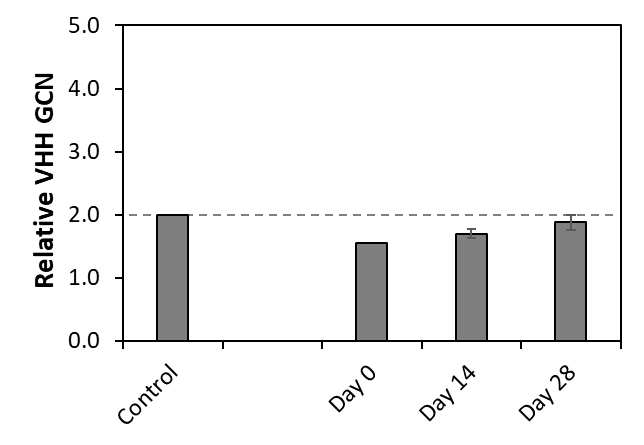


**Figure S4. Genetic stability of the VHH expression cassette in retentostat cultures.** Bars represent the relative VHH gene copy number measured by qPCR. Genomic DNA of the control was isolated from an overnight culture grown on selective media, while genomic DNA of retentostat samples was isolated from samples taken on days 0, 14, and 28 of the retentostat phase.

**
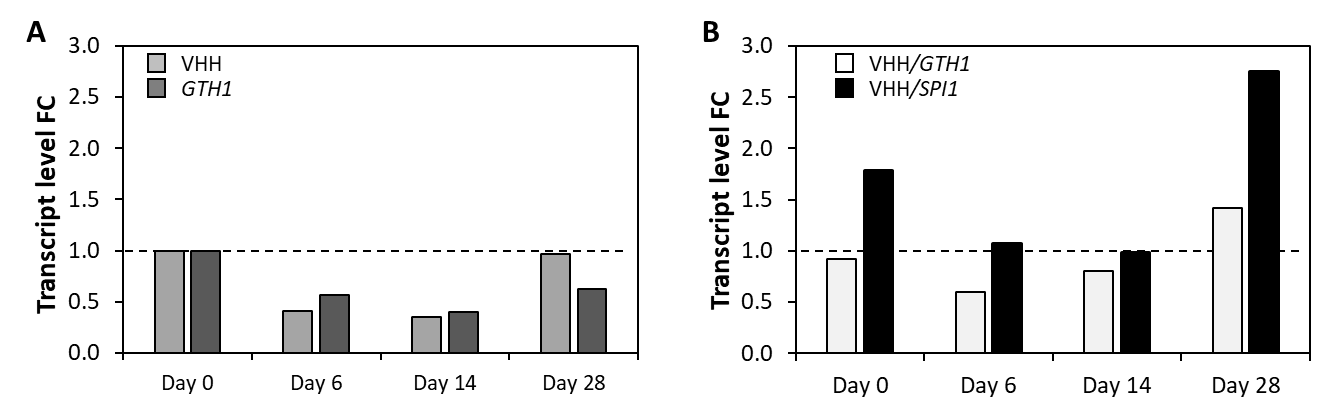
**

**Figure S5. Transcript level profile and relative transcriptional strength of VHH in retentostat cultures. A)** Bars represent the relative VHH and *GTH1* transcript level fold-change between samples taken at time point 0 when retentostats were initiated (set to 1) and later sampling points throughout the retentostat phase. Transcript levels were normalized to *ACT1*. **B**) Bars represent the absolute transcript level fold-change between VHH and *GTH1* as well as VHH and *SPI1* at corresponding sampling points. Absolute transcript level determination was done using a standard curve of the purified PCR product of the respective gene.

**Table S2: Macromolecular biomass composition over a wide range of μ.**

| **Cultivation mode** | **Day** | **μ**  **[h^-1^]** | **Carbohydrate**  **[%]** | **Protein**  **[%]** | **RNA**  **[%]** | **DNA**  **[%]** | **Lipid**  **[%]** |
| --- | --- | --- | --- | --- | --- | --- | --- |
| **Chemostat** | - | 0.100 | 45.5 ± 0.5 | 32.3± 0.2 | 4.6 ± 0.7 | 0.7 ± 0.0 | 2.7 ± 0.1 |
| **Chemostat** | 0 | 0.025 | 52.0 ± 1.2 | 29.8 ± 1.0 | 3.8 ± 0.1 | 0.7 ± 0.0 | 1.7 ± 0.1 |
| **Retentostat** | 6 | 0.0022 | 53.9 ± 0.9 | 27.5 ± 0.6 | 3.7 ± 0.1 | 0.5 ± 0.0 |  |
| **Retentostat** | 14 | 0.0010 | 55.4 ± 1.7 | 28.3 ± 0.8 | 3.6 ± 0.0 | 0.4 ± 0.0 | 2.2 ± 0.1 |
| **Retentostat** | 28 | 0.00047 | 54.1 ± 1.1 | 27.5 ± 0.7 | 3.4 ± 0.1 | 0.4 ± 0.0 | 3.2 ± 0.2 |

**Table S3: Elemental biomass composition over a wide range of μ.**

| **Cultivation mode** | **μ**  **[h^-1^]** | **C**  **[%]** | **H**  **[%]** | **N**  **[%]** | **O**  **[%]** | **S**  **[%]** | **P**  **[%]** |
| --- | --- | --- | --- | --- | --- | --- | --- |
| **Chemostat** | 0.100 | 42.7 ± 0.2 | 6.7 ± 0.1 | 8.3 ± 0.9 | 36.3 ± 0.2 | 0.23 ± 0.01 | 2.15 ± 0.04 |
| **Chemostat** | 0.025 | 42.1 ± 0.1 | 6.2 ± 0.8 | 7.1 ± 0.3 | 37.8 ± 0.2 | 0.21 ± 0.01 | 1.86 ± 0.15 |
| **Retentostat** | 0.0022 | 41.6 ± 0.3 | 6.7 ± 0.1 | 6.9 ± 0.1 | 39.0 ± 0.2 | 0.19 ± 0.01 | 2.02 ± 0.12 |
| **Retentostat** | 0.0010 | 41.6 ± 0.2 | 6.7 ± 0.0 | 7.0 ± 0.2 | 38.6 ± 0.2 | 0.20 ± 0.02 | 2.11 ± 0.13 |
| **Retentostat** | 0.00047 | 41.8 ± 0.3 | 6.7 ± 0.1 | 6.8 ± 0.1 | 38.0 ± 0.2 | 0.18 ± 0.01 | 2.20 ± 0.07 |

**Table S4. *p*-values of pairwise comparisons of the total carbohydrate, protein, RNA, DNA, and lipid content measurements based on the Student's t-test.** Statistically significant changes (*p*-value < 0.05) are highlighted in light green.

**
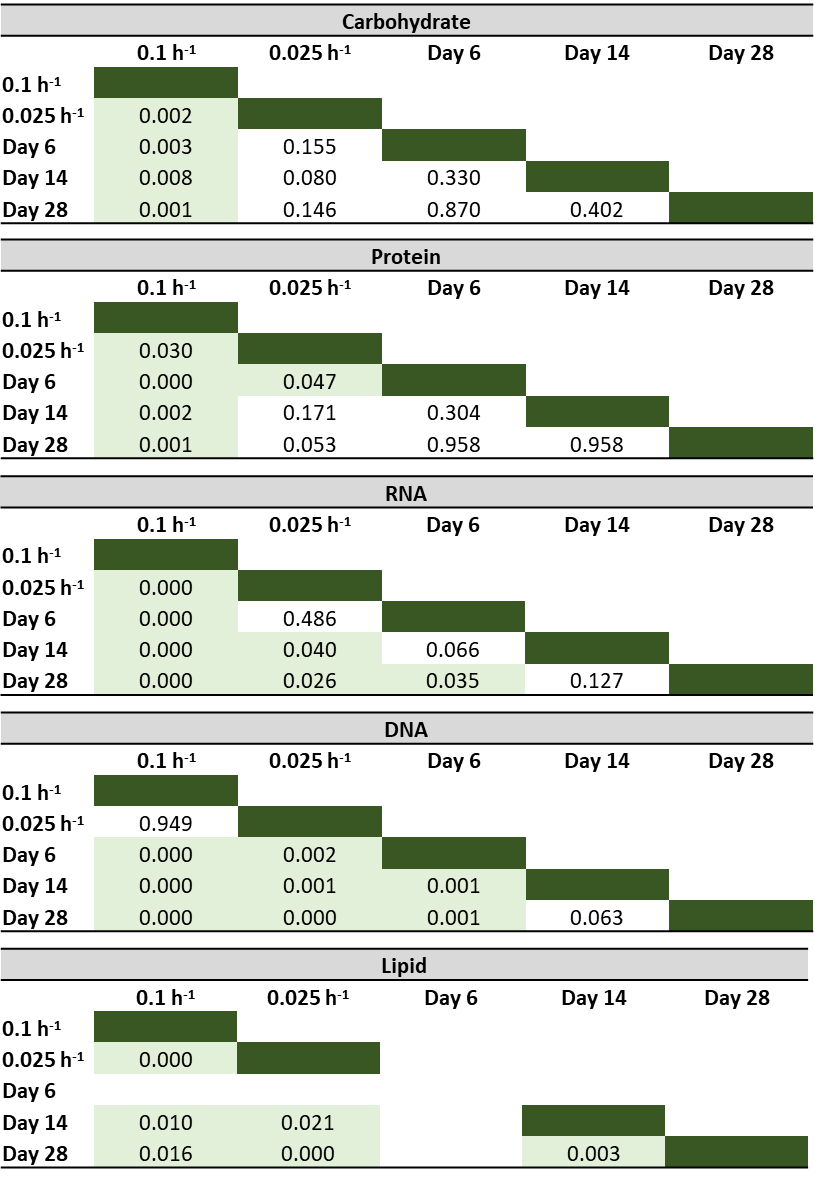
**

**
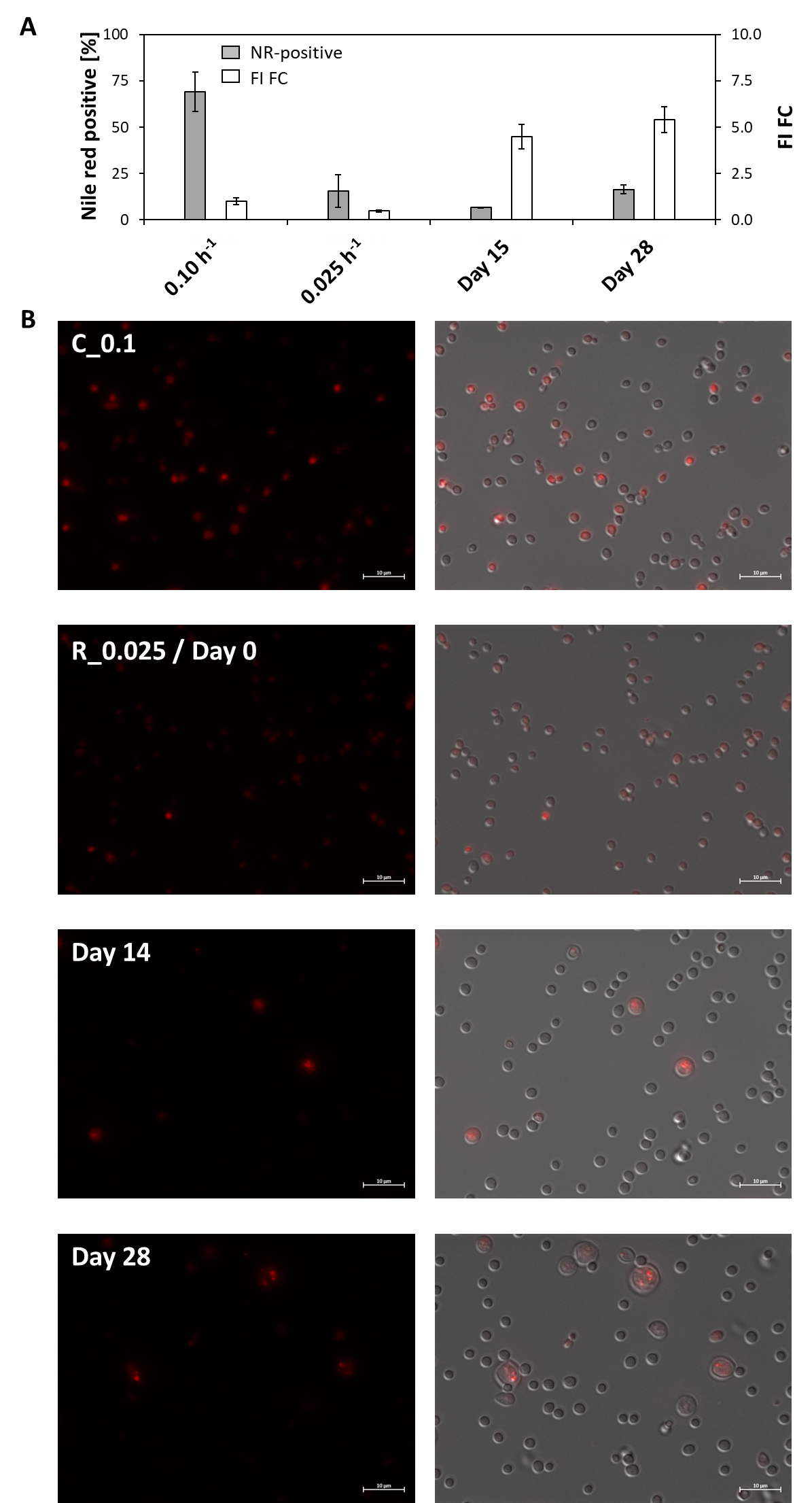
**

**Figure S6. Flow cytometry and fluorescence microscopy of chemostat and retentostat samples stained with Nile red. A)** Shown are the average fraction of Nile red (NR) positive cells according to flow cytometry analysis and the fold-change of the fluorescence intensity of the respective NR-positive fraction relative to the 0.1 h^-1^ setpoint. **B)** Fluorescence microscopy of NR-stained cells.

**Table S5**: Comparison between the amino acid composition in this study at *D*=µ=0.1 h^-1^ and values obtained by Carnicer et al. (2009) for Fab-expressing K*. phaffii* X-33 at 21% oxygen and the same *D*.

| **% mol mol^-1^** | **Fab-expressing X-33**  **(*D*=µ=0.1 h^-1^; 21% O_2_)** | **VHH-expressing CBS2612 Δ*flo8* (*D*=µ=0.1 h^-1^; 21% O_2_)** |
| --- | --- | --- |
| Alanine | 10.40 | 8.75 |
| Arginine | 7.04 | 6.55 |
| AsX | 8.82 | 10.05 |
| Cysteine | 0.15 | 1.27 |
| GlX | 17.81 | 16.59 |
| Glycine | 6.86 | 7.03 |
| Histidine | 1.89 | 1.69 |
| Isoleucine | 4.64 | 3.88 |
| Leucine | 6.96 | 7.06 |
| Lysine | 6.41 | 6.21 |
| Methionine | 0.79 | 1.27 |
| Phenylalanine | 3.20 | 3.18 |
| Proline | 3.83 | 4.39 |
| Serine | 6.26 | 7.28 |
| Threonine | 5.88 | 5.93 |
| Tryptophan* | 1.40 | 1.40 |
| Tyrosine | 2.16 | 2.49 |
| Valine | 5.88 | 4.99 |

*Tryptophan was not measured in the present study, therefore the value from Carnicer et al. (2009) was used and the data corrected accordingly.

**
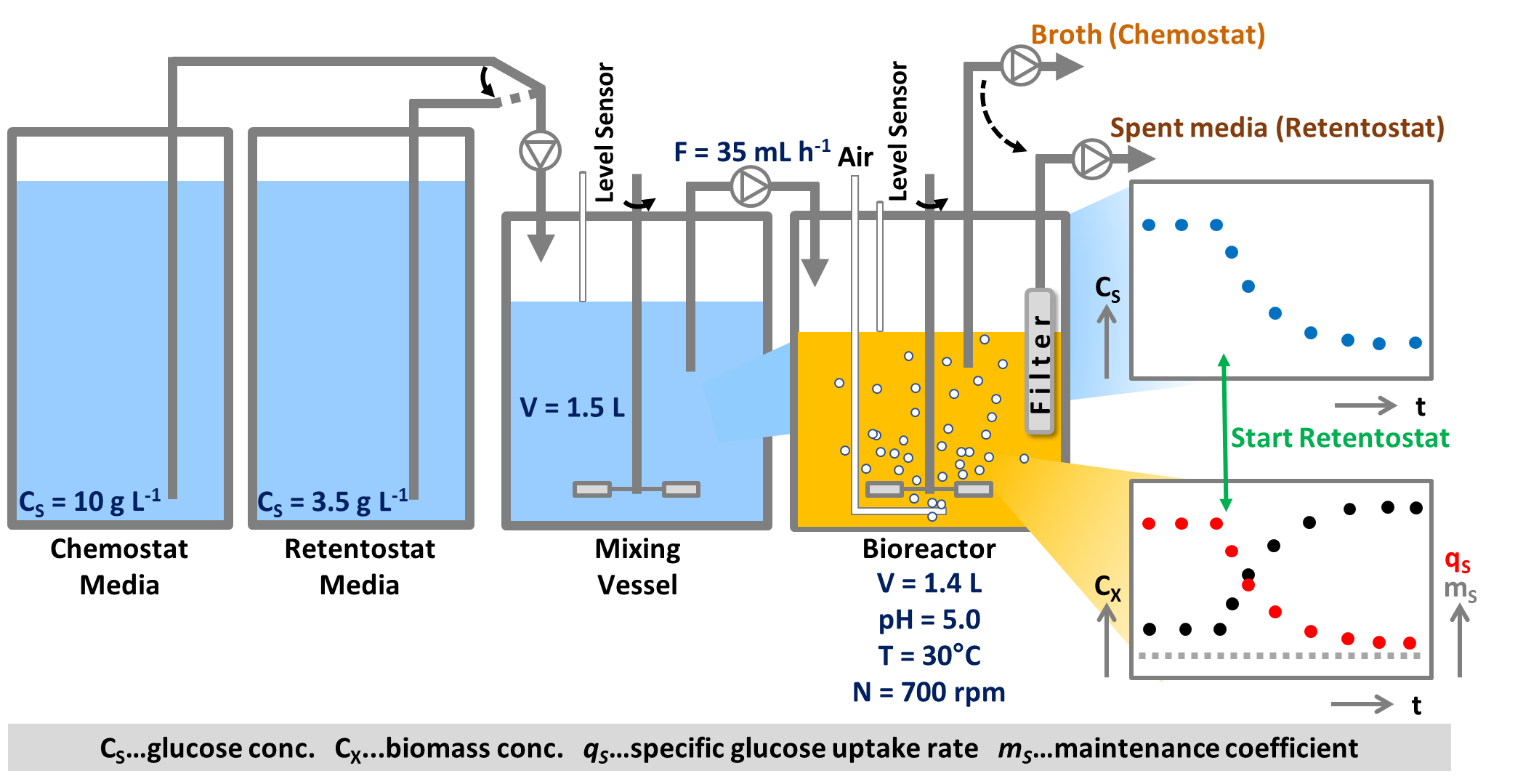
 Figure S7. Schematic representation of the retentostat setup and growth kinetics in retentostat.** Retentostat cultures were initiated from chemostat cultures operated at a dilution rate (*D*) of 0.025 h^-1^ by re-directing the effluent through a filter device. Upon initiation of the retentostat phase, a glucose gradient was established (C_S_ decreases from 10 to 3.5 g L^-1^) by means of a mixing vessel. The working volume of the mixing and bioreactor vessel were kept constant by means of a level sensor. This means that for the mixing vessel the feed pump would start feeding media from the chemostat or retentostat media reservoir once the level sensor is no longer touching the surface, while for the bioreactor the harvest pump would switch on if the level sensor circuit is closed.

**Supporting Material and Methods.**

**Protease activity** in culture supernatants was measured employing the Pierce™ Colorimetric Protease Assay Kit (Thermo Scientific™) according to the suppliers’ instructions.

**Genomic DNA isolation** for gene copy number (GCN) determination was done by employing the Wizard® Genomic DNA Purification Kit (Promega) according to the supplier’s instructions. DNA purity and concentration were analyzed on a NanoDrop 2000 (Thermo Scientific).

**cDNA synthesis** for quantitative RT PCR was done employing the Biozym cDNA Synthesis Kit in combination with Oligo d(T)_23_ VN primer (NEB) according to the supplier’s instructions.

**qPCR.** Primers for qPCR for transcript level and GCN determination are shown in **Table S5**. *ACT1*-specific primers were used for normalization. For analysis, appropriate amounts of genomic DNA or cDNA were mixed according to the supplier’s recommendations with water, primers, and 2x qPCR S’Green BlueMix (Biozym Blue S’Green qPCR Kit) and measured in a real-time PCR cycler (Rotor-Gene, Qiagen). All samples were measured at least in technical triplicates. Data analysis was performed with the Rotor-Gene software, employing the Comparative Quantitation method for gene copy analysis and the Delta-Delta-Ct-Method for transcript level analysis. For the latter, purified PCR products of known concentration were used as standards.

**Table S6**: **qPCR primers for transcript and GCN analysis**

**Primer Sequence**

*ACT1*_fw CCTGAGGCTTTGTTCCACCCACT

*ACT1*_bw GGAACATAGTAGCAC CGGCATAACGA

*GTH1*_fw ATGGCACGAACATAGCCAAGGCA

*GTH1*_bw TCGTGTGCTCCTTTGCTCCAAC

*SPI1*_fw GACTGTCCTTGCACCATCTCCAA

*SPI1*_bw GGTAGAGGTAGATGGCACGTGAGT

VHH_fw TGTAACGTGAATGTCGGATTTG

VHH_bw TAGTGATGGTGGTGGTGATG

**Protein amino acid composition analysis**: For hydrolysis followed by GC-MS/MS analysis, the protocol published in Szeliová et al. (2020) was used. The method deviates in the selection of the quantifier and qualifier transitions, as the selection of these transitions was adapted to the *K. phaffii* sample matrix. The transitions for the quantifier and qualifier and the corresponding internal standards are listed in **Table S6**. Likewise, it was reevaluated which hydrolysis method was employed for the final data evaluation, as for a number of AA, both hydrolysis methods are principally suitable.

As the signal-to-noise ratio was higher in LC-MS/MS than in GC-MS/MS, quantification of arginine and histidine was performed via LC-MS/MS (Thermo Scientific CTC PAL autosampler, Accela 1250 pump, TSQ Vantage MS/MS). The following MS settings were employed for the heated electrospray source: capillary temperature 350°C, spray voltage 3300 V, sheath gas pressure 60 psig, auxiliary gas pressure 15 psig, vaporizer temperature 350°C, scan polarity positive. An XBridge Amide analytical column (150 x 2.1 mm, 3.5 µm particle size, Waters, Milford, USA) was used for hydrophilic interaction liquid chromatography (HILIC) gradient separation employing a column temperature of 40°C and an injection volume of 5 µL. Solvent A was acetonitrile, and solvent B was 10 mmol L^-1^ ammonium formate with a mobile phase gradient of 10-70% (v/v) B applied between 1 and 12 min at a flow rate of 350 µL min^-1^.

**Table S6: Quantifier and qualifier transitions and their corresponding internal standard (ISTD) transitions for protein amino acid analysis and choice of hydrolysis technique.**

| **Compound** | **Type of transition** | **tr**  **(min)** | **Precursor Ion (m/z)** | **Product Ion (m/z)** | **Collision Energy (V)** | **Resolution** | **Hydrolysis** |
| --- | --- | --- | --- | --- | --- | --- | --- |
| **GC-MS/MS** |  |  |  |  |  | **Resolution MS1, MS2** |  |
| Alanine | Quantifier* | 4.02 | 232.2 | 147.1 | 15 | Unit | MSA |
|  | ISTD Quant | 4.02 | 235.2 | 147.1 | 15 | Unit |  |
|  | Qualifier* | 4.02 | 260.2 | 232.2 | 5 | Unit |  |
|  | ISTD Qual | 4.02 | 264.2 | 235.2 | 5 | Unit |  |
| Glycine | Quantifier* | 4.12 | 246.1 | 218.2 | 5 | Unit | MSA |
|  | ISTD Quant | 4.13 | 249.1 | 220.2 | 5 | Unit |  |
|  | Qualifier* | 4.13 | 218.2 | 147.1 | 15 | Unit |  |
|  | ISTD Qual | 4.13 | 220.2 | 147.1 | 15 | Unit |  |
| Valine | Quantifier* | 4.60 | 260.2 | 147.1 | 15 | Unit | HCl |
|  | ISTD Quant | 4.60 | 265.2 | 147.1 | 15 | Unit |  |
|  | Qualifier* | 4.60 | 186.2 | 73.0 | 15 | Unit |  |
|  | ISTD Qual | 4.60 | 191.2 | 73.0 | 15 | Unit |  |
| Leucine | Quantifier* | 4.78 | 302.2 | 274.2 | 10 | Unit | HCl |
|  | ISTD Quant | 4.78 | 309.2 | 280.3 | 10 | Unit |  |
|  | Qualifier* | 4.78 | 302.2 | 200.2 | 15 | Unit |  |
|  | ISTD Qual | 4.78 | 309.2 | 206.2 | 15 | Unit |  |
| Isoleucine | Quantifier* | 4.78 | 302.2 | 274.2 | 10 | Unit | HCl |
|  | ISTD Quant | 4.78 | 309.2 | 280.3 | 10 | Unit |  |
|  | Qualifier* | 4.78 | 274.3 | 147.1 | 15 | Unit |  |
|  | ISTD Qual | 4.78 | 280.3 | 147.1 | 15 | Unit |  |
| Proline | Quantifier* | 5.20 | 258.3 | 147.1 | 15 | Unit | MSA |
|  | ISTD Quant | 5.20 | 263.3 | 147.1 | 15 | Unit |  |
|  | Qualifier* | 5.20 | 286.2 | 258.2 | 10 | Unit |  |
|  | ISTD Qual | 5.20 | 292.2 | 263.2 | 10 | Unit |  |
| Serine | Quantifier* | 6.25 | 390.2 | 362.3 | 15 | Unit | MSA |
|  | ISTD Quant | 6.25 | 394.2 | 365.3 | 15 | Unit |  |
|  | Qualifier* | 6.25 | 390.2 | 258.2 | 15 | Unit |  |
|  | ISTD Qual | 6.25 | 394.2 | 262.2 | 15 | Unit |  |
| Threonine | Quantifier* | 6.42 | 404.4 | 376.4 | 15 | Wide | MSA |
|  | ISTD Quant | 6.42 | 409.2 | 380.4 | 15 | Wide |  |
|  | Qualifier* | 6.42 | 376.4 | 244.2 | 10 | Wide |  |
|  | ISTD Qual | 6.42 | 380.2 | 248.2 | 10 | Wide |  |
| Phenylalanine | Quantifier* | 6.88 | 336.3 | 308.2 | 10 | Unit | HCl |
|  | ISTD Quant | 6.88 | 346.2 | 317.2 | 10 | Unit |  |
|  | Qualifier* | 6.88 | 336.3 | 204.1 | 15 | Unit |  |
|  | ISTD Qual | 6.88 | 346.2 | 214.1 | 15 | Unit |  |
| Aspartate | Quantifier* | 7.20 | 418.4 | 244.2 | 10 | Unit | MSA |
|  | ISTD Quant | 7.20 | 423.3 | 247.2 | 10 | Unit |  |
|  | Qualifier* | 7.20 | 418.4 | 390.3 | 10 | Unit |  |
|  | ISTD Qual | 7.20 | 423.3 | 394.3 | 10 | Unit |  |
| Glutamate | Quantifier* | 7.95 | 432.4 | 147.1 | 30 | Unit | MSA |
|  | ISTD Quant | 7.95 | 438.4 | 147.1 | 30 | Unit |  |
|  | Qualifier* | 7.95 | 432.4 | 272.2 | 15 | Unit |  |
|  | ISTD Qual | 7.95 | 438.4 | 277.2 | 15 | Unit |  |
| Methionine (quantified via methionine sulfone) | Quantifier* | 8.10 | 352.2 | 244.2 | 15 | Unit | HCl |
|  | ISTD Quant | 8.10 | 358.2 | 248.2 | 15 | Unit |  |
|  | Qualifier* | 8.10 | 352.2 | 324.2 | 5 | Unit |  |
|  | ISTD Qual | 8.10 | 358.2 | 329.2 | 5 | Unit |  |
| Cysteine (quantified via cysteic acid) | Quantifier* | 8.75 | 454.3 | 258.2 | 10 | Unit | HCl |
|  | ISTD Quant | 8.75 | 458.3 | 262.2 | 10 | Unit |  |
|  | Qualifier* | 8.75 | 426.3 | 147.1 | 20 | Unit |  |
|  | ISTD Qual | 8.75 | 429.2 | 147.1 | 20 | Unit |  |
| Lysine | Quantifier* | 8.76 | 329.3 | 198.0 | 10 | Unit | MSA |
|  | ISTD Quant | 8.76 | 336.3 | 204.0 | 10 | Unit |  |
|  | Qualifier* | 8.76 | 431.3 | 300.3 | 10 | Unit |  |
|  | ISTD Qual | 8.76 | 439.3 | 307.3 | 10 | Unit |  |
| Tyrosine | Quantifier* | 10.68 | 466.3 | 438.4 | 30 | Unit | MSA |
|  | ISTD Quant | 10.68 | 476.4 | 447.4 | 30 | Unit |  |
|  | Qualifier* | 10.68 | 466.3 | 147.1 | 30 | Unit |  |
|  | ISTD Qual | 10.68 | 476.4 | 147.1 | 30 | Unit |  |
| **LC-MS/MS** |  |  |  |  |  | **Q1 Peak Width** |  |
| Arginine | Quantifier | 6.92 | 175.1 | 116.0 | 13 | 0.7 | MSA |
|  | ISTD Quant | 6.92 | 185.1 | 122.0 | 13 | 0.7 |  |
|  | Qualifier | 6.92 | 175.1 | 70.0 | 24 | 0.7 |  |
|  | ISTD Qual | 6.92 | 185.1 | 77.0 | 24 | 0.7 |  |
| Histidine | Quantifier | 7.17 | 156.1 | 110.0 | 13 | 0.7 | MSA |
|  | ISTD Quant | 7.17 | 165.1 | 118.0 | 13 | 0.7 |  |
|  | Qualifier | 7.17 | 156.1 | 83.0 | 25 | 0.7 |  |
|  | ISTD Qual | 7.17 | 165.1 | 89.0 | 25 | 0.7 |  |

* The average of quantifier and qualifier was used for data evaluation.

**Lipid composition analysis.** Reference standards were obtained from Avanti (Alabaster, USA) and Sigma Aldrich (Vienna, Austria). All chemicals were of LC-MS grade and ordered at Fisher Scientific (Vienna, Austria), VWR International (Vienna, Austria), or Sigma Aldrich (Vienna, Austria). ^13^C-labeled *K. phaffii* was obtained by Isotopic Solutions (Vienna, Austria).

For extraction, a dried cell suspension of ^13^C-labeled *Pichia pastoris* (1 g ±0.2 g equals 1.5x10^10^ cells) was washed with 20 mL of ammonium bicarbonate (ABC) buffer (150 mM) to remove salts from the matrix. It was centrifuged for 10 min at 1000 rcf, and the supernatant was descanted and discarded. The pellet was again dissolved in 3 mL of ABC buffer and mixed vigorously to get an estimated cell density of 5x10^9^ cells/mL. During all steps, the ISTD was stored on ice if possible.

To the sample cell pellet (5-19 mg dry mass; ~2.8-8.5x10^8^cells), 100 µL of the ISTD cell mix was added for a similar cell density. The cells were transferred into 2 mL polypropylene tubes, which were suitable for mechanical disruption. Glass beads have already been inserted into the tubes with an amount corresponding to a liquid equivalent of 400 μL. 900 µL of MeOH was added, and each tube was shaken three times for 10 s at full speed, respectively (*Peqlab Minilys)* and between each lysis step, the sample was cooled on ice for one minute. For the extraction, the homogenate was transferred into 10 mL glass tubes with Teflon lids. 700 µL MeOH and 3.3 mL CHCl_3_ were used for transfer. The extracts were shaken on ice for 1.5 h at 200 rpm. Afterward, they were removed from the ice, and 1.6 mL of ABC buffer (150 mM) was added and shaken to initiate phase separation. After the two phases were clear, the upper phase was removed until the lower lipid-containing phase was free of any upper layer. 200-µL aliquots of the organic lower phase were put into glass vials, evaporated to dryness under nitrogen, and stored at -20°C until further treatment. The samples were re-suspended in 200 µL of IPA for LC-MS injection.

For analysis, an Acquity HSS T3 (2.1 mm x 150 mm, 1.8 µm, Waters) equipped with a VanGuard Pre-column (2.1 x 5 mm, 100 Å, 1.8 µm, Waters) was used on a Vanquish™ Horizon HPLC (Thermo Scientific) coupled to a high field Q Exactive HF quadrupole-Orbitrap mass spectrometer (Thermo Scientific). As eluents Acetonitrile (ACN)/H_2_O (3:2, v/v) (eluent A) and IPA/ACN (9:1, v/v) (eluent B), both containing 0.1% formic acid and 10 mM ammonium formate, were used. The flow rate was set to 250 µL min^-1^ and the column temperature to 40 °C. The following gradient was applied: start at 30% B, 0−2.0 min ramp to 43% B, 2.0−2.1 min ramp to 55% B, 2.1−12.0 min ramp to 65% B, 12.0-18.0 min ramp to 85% B, 18.0-20.0 min ramp to 100% B, 20.0-25.0 min 100% B, 25.1 min fast switch to 30% B and equilibrated at the starting conditions 25.1-30.0 min 30% B. The injector needle was washed with 75% IPA, 25% H_2_O, and 0.1% formic acid for 5 s prior to each injection. The temperature of the autosampler was set to 10°C, and the injection volume was 2 µL. The ESI source parameters were the following: sheath gas 30(+)/ 35(-), auxiliary gas 5(+)/ 10(-), spray voltage 3.5 kV(+)/ 2.8 kV(-), capillary temperature 220°C(+)/ 250°C(-), S-Lens RF level 45, and auxiliary gas heater 300 °C. Spectral data was acquired in profile mode.

The full MS runs for quantification were acquired in the range of *m/z* 200–2000 at a resolution of 120,000, an AGC target at 1e6, and a maximum IT of 200 ms. Data-dependent MS2 (ddMS2) fragmentation spectra were acquired for identification. A Top8 method with a normalized collision energy of 25(+)/28(-) and an isolation window of 1 Da was applied. The resolution in the MS2 was set to 30,000, the AGC target to 2e5 (minimum 8e3), and the max IT to 60 ms. The dynamic exclusion of triggered *m/z* was set to 15 s. Both, an inclusion and an exclusion list were used for the possible lipids in yeast and the background compounds identified in a blank run, respectively.

Lipid identification was performed with LipidSearch 4.2 from Thermo Scientific, in which the following filters were applied: RT tolerance 0.25 min, m-score threshold 5, ID quality filter A,B,C (D- only for free fatty acids and cardiolipins), calculate unassigned peak area TRUE and toprank filter TRUE. The external multipoint calibration internal standardized was performed in R/R studio.

**Elemental analysis.** Lyophilized biomass (about 6 mg) was weighed on a Satorius microbalance, and a FlashSmartTM Elemental Analyzer was used to determine the CHNS/O composition. Phosphorus was quantified by the ammonium heptamolybdate technique.

For the determination of CHNS, the configuration included an additional built-in quartz tube in the reactor furnace and a reactor attachment made of copper oxide and elemental copper connected to a gas chromatographic column (CC1, PTFE material, 2m diameter, 6x5 mm) and a thermal conductivity detector (TCD). For the determination of oxygen, the configuration consisted of a quartz reactor with a nickel-coated carbon catalyst and fine-grain quartz elements, no oxygen injection (reactor 2), and an adsorption filter consisting of soda lime (CO2 trap) and magnesium perchlorate as a water trap. Chromatographic separation was done by a molecular sieve column, and detection was done by a TDC.

Phosphor was measured by colorimetric determination using the ammonium heptamolybdate method after nitric and perchloric acid digestions at 220°C.

**Yeast lipid droplet staining with Nile red.** Yeast cells were diluted to an OD_600_ of 4.0 with PBS, centrifuged at 2000 rpm for 3 min and resuspended in 0.4 mL of a 1 μM Nile red solution. After incubation at 30°C for 10 min the cells were washed and resuspended in 0.5 mL of PBS. For cytometry, cells were further diluted to an OD_600_ of 0.4 in PBS. Cytometric analysis was done on a Cytoflex and fluorescence microscopy on a Zeiss Axiom employing filter cube N2.1.
